# Insights into Molecular Interactions and Biological Effect of Natural Stilbenoids at the TRPA1 Ion Channel

**DOI:** 10.1002/cmdc.202400501

**Published:** 2024-11-20

**Authors:** Atefeh Saadabadi, Marja Rantanen, Parthiban Marimuthu, Ari‐Pekka Koivisto, Patrik C. Eklund, Outi M. H. Salo‐Ahen

**Affiliations:** ^1^ Pharmaceutical Sciences Laboratory Faculty of Science and Engineering Åbo Akademi University Tykistökatu 6 Turku 20520 Finland; ^2^ Laboratory of Molecular Science and Engineering Faculty of Science and Engineering Åbo Akademi University Henrikinkatu 2 Turku 20500 Finland; ^3^ Structural Bioinformatics Laboratory Faculty of Science and Engineering Åbo Akademi University Tykistökatu 6 Turku 20520 Finland; ^4^ Pain Therapy Area R&D, Orion Pharma Tengströminkatu 8 Turku 20360 Finland; ^5^ Center for Global Health Research Saveetha Medical College Saveetha Institute of Medical and Technical Sciences Chennai 602 105 India

**Keywords:** Ion channels, Molecular docking, Molecular dynamics, Natural stilbenoids, TRPA1 modulators

## Abstract

Natural stilbenoids, polyphenolic compounds notably found in Scots pine and Norway spruce, have been shown to exhibit analgesic and anti‐inflammatory effects through the TRPA1 channel, making them promising hits for the development of novel agents to treat inflammatory diseases and pain. In this study, we computationally investigated the putative binding sites of natural stilbenoids at the TRPA1 channel. Specifically, we employed molecular docking and MD simulation approaches to explore three known ligand binding sites at TRPA1. Furthermore, the biological effect of the studied compounds on TRPA1 was assessed *in vitro* using a fluorescent imaging plate reader (FLIPR™) calcium assay. Our modeling results suggest the stilbenoids exhibit higher affinity to the two agonist binding sites than the antagonistic site. Consistent with this, the *in vitro* results showed that the stilbenoids act as moderate TRPA1 channel agonists and likely inhibit the channel through a desensitization mechanism rather than act as pure TRPA1 antagonists. Additionally, our bias‐force pulling simulations proposed an additional binding pocket for the natural stilbenoids that is distinct from the known ligand binding sites at TRPA1. The results of the study give useful insights into structure‐based design and development of novel therapeutic TRPA1 modulators.

## Graphical abstract

Binding of the natural stilbenoids, isolated from Norway spruce and Scots pine, was analyzed at three known binding sites of transient receptor potential ankyrin 1 (TRPA1) using molecular docking and molecular dynamics (MD) simulations. Furthermore, bias‐force pulling simulations predicted a putative novel binding pocket for the stilbenoids, distinct from the previously reported ligand‐binding sites.

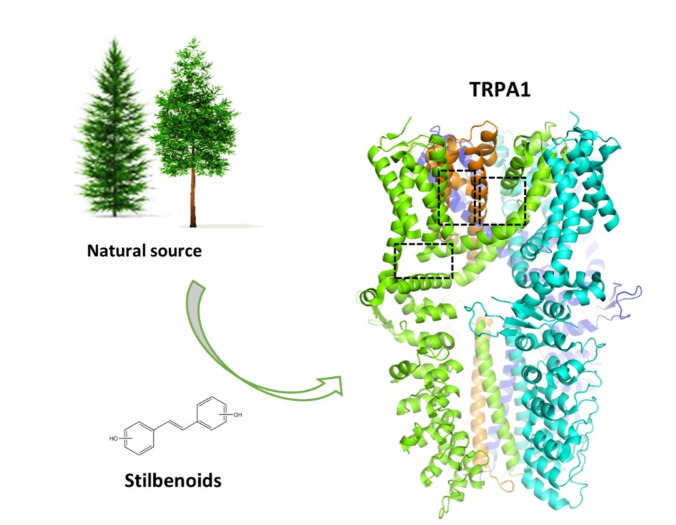

## Introduction

Stilbenoids comprise a class of polyphenolic compounds that in the last decades have captured the intense attention of many research groups worldwide for their diverse biological activities. So far, they have been shown to possess health‐promoting properties including antioxidant, anticancer, antidiabetic, anti‐inflammatory, antimicrobial and antifungal activity, as well as neuroprotection, cardioprotection, and inhibition of melanin synthesis in hyperpigmentation disorders.^[1]^ Stilbenoids’ molecular mechanism (particularly that of resveratrol) in many pathological conditions has been intensively studied in recent years, and the mechanistic details of the compounds’ action on specific target proteins have been elucidated.[^2^, ^3^, ^4^, ^5^] However, much remains to be solved. For example, the structural insights into stilbenoids’ interactions with the transient receptor potential (TRP) family of ion channels,^[6]^ specifically TRPA1 (ankyrin1), have remained a mystery that we seek to shed light upon in this study.

TRPA1, a non‐selective cation channel, is gated by several endogenous mediators produced at sites of tissue injury and inflammation, including oxidative stress components. It is also responsive to exogenous stimuli such as mechanical, thermal, and chemical factors. As this channel contributes to different acute and chronic pain and inflammatory pathways, it is considered a promising target for the development of analgesic and anti‐inflammatory agents.[^7^, ^8^, ^9^] However, despite remarkable efforts in generation of TRPA1 modulators, so far, none of the candidate compounds has reached the market. However, the successful completion of the Phase I clinical trials and reaching Phase II by potent hTRPA1 antagonistic compounds such as GRC‐17536^[10]^ and LY3526318[^11^, ^12^] (both now discontinued) or GDC‐6599^[13]^ (on‐going) offers hope that TRPA1 modulators could eventually be developed as viable drugs. In what follows, we will give a detailed overview of the so far identified ligand binding sites at TRPA1 and describe the TRPA1‐related activities previously reported for stilbenoids to give the reader a proper background for understanding the context and rationale of this work.

**Figure 1:**
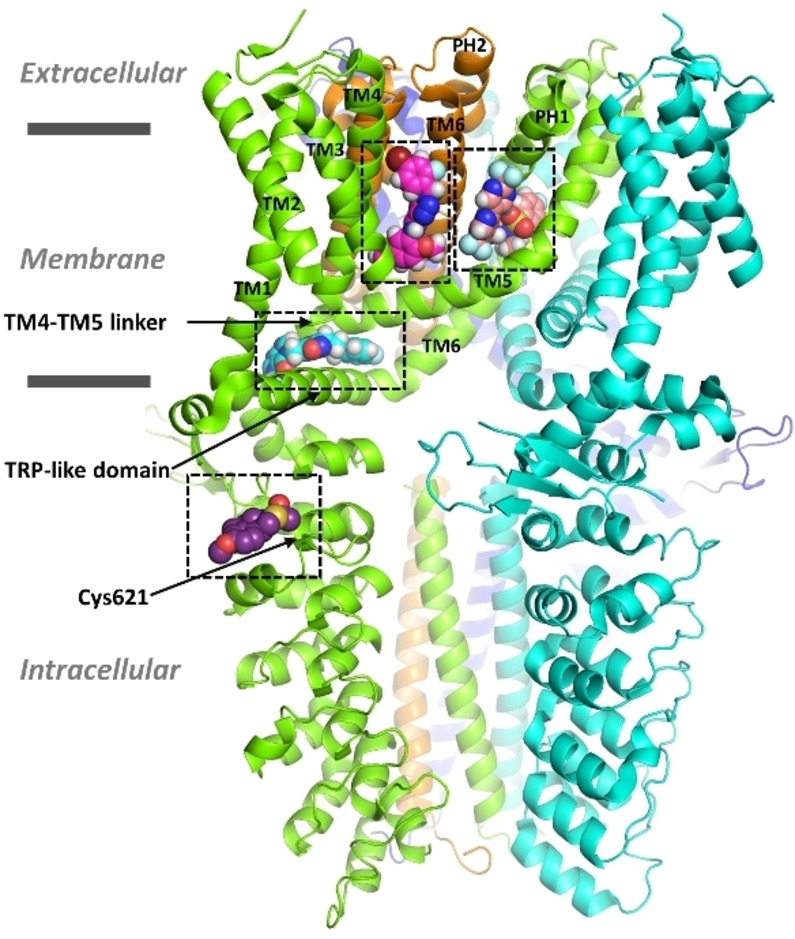
Human TRPA1 (hTRPA1) structure (cartoon) and the binding sites of four known TRPA1 ligands (in spheres). Ligand atom color code: nitrogen – blue; oxygen – red; sulfur – gold; fluorine – light blue; bromine – dark red; hydrogen – white; carbon – salmon (GDC‐0334, antagonist), magenta (GNE‐551, agonist), cyan (compound [cpd] 21, antagonist) and purple (JT010, agonist). The ligand binding sites are delineated in black dashed lines and were located by superposing the cryo‐EM structures with PDB IDs 6WJ5, 6X2J, 7JUP and 6PQO, respectively, in PyMOL v. 2.5.5. The TM domains are labelled in the binding site‐forming subunits. The four subunits are presented in green, cyan, orange, and blue cartoon.

TRPA1 is a homo‐tetrameric ion channel that comprises intracellular N‐ and C‐terminal domains and six transmembrane (TM) domains (Figure 1). TM1–TM4 domains form the voltage‐sensor‐like domain (VSLD) whereas TM5 and TM6 domains together with the loop that connects them form the channel pore.^[14]^ The pore‐forming loop contains two alpha helical structures, pore helix (PH) 1 and PH2. The upper gate and selectivity filter of the pore is formed by one residue, Asp915 that resides in the loop portion connecting the pore helices. The lower gate of the channel pore, on the other hand, is formed by two hydrophobic residues at the end of TM6, Ile957 and Val961.

**Figure 2:**
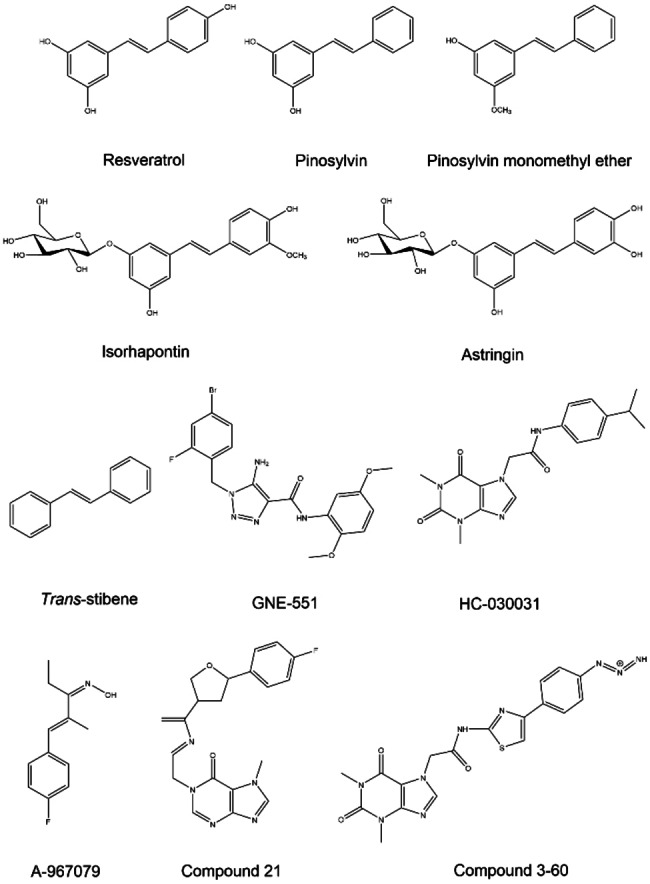
The structures of the natural stilbenoids and the reference compounds used in this study.

The channel can be modulated with various electrophilic and non‐electrophilic chemicals (see Supporting Information, Figure S1 and Table S1). The binding pocket for electrophilic TRPA1 agonists such as allyl isothiocyanate (AITC), methylglyoxal, 2‐chloro‐*N*‐(4‐(4‐methoxyphenyl)thiazol‐2‐yl)‐*N*‐(3‐methoxypropyl)‐acetamide (JT010) and benzyl isothiocyanate (BITC) that covalently (reversibly or irreversibly) modify the channel is located in the N‐terminal domain nearby (or possibly even formed by) amino acids Cys621, Cys641 and Cys665 (Figure 1).[^15^, ^16^] In contrast, two other agonistic sites for non‐electrophilic compounds are situated within the TM domains. The first site, recognized as a binding pocket for menthol,^[17]^ general anesthetics (propofol and isoflurane)^[18]^ and anethole^[19]^ is lined by TM5 and PH1 from one subunit, as well as TM6 from the adjacent subunit. Residues Ser873 and/or Thr874 at that site have been identified as critical for ligand sensitivity in human TRPA1 (hTRPA1).^[16]^ Interestingly, isoflurane antagonizes the inhibitory effect of A‐967079 (a potent and selective TRPA1 antagonist; Figure 2) in rat TRPA1 (rTRPA1) through a competitive mechanism,^[18]^ suggesting that the antagonist may at least partially share the binding site with general anesthetics. Amino acids Met912 and Met953 (corresponding to Met915, Met956 in rTRPA1), in addition to Ser873, have also been shown to be essential for the activity of general anesthetics.^[18]^ Moreover, menthol can exhibit bimodal activity, leading to channel activation at low concentrations and inhibition at higher concentrations in mouse TRPA1 (mTRPA1).^[20]^ Residue Gly878 (at TM5) in mTRPA1 (corresponding to Val875 in hTRPA1) was identified responsible for this species‐specific gating and sensitivity to menthol.^[17]^ Furthermore, Memon et al.^[19]^ demonstrated that repeated application of anethole desensitizes hTRPA1 channel over time. Unlike AITC, anethole does not evoke detectable nocifensive behavior in animals, being thus a less‐irritating agonist compared to AITC.

The second agonistic site for non‐electrophilic compounds was recently identified for GNE‐551 (a potent and selective TRPA1 agonist) (Figures 1&2). It is situated within a pocket between TM4 (with key residues: Aln836, Tyr840 and Phe841) of one subunit and TM5 (Ser887) and TM6 (Gln940 and Phe947) of the adjacent subunit.^[21]^ This pocket has also been observed in rat and squirrel TRP vanilloid 1 (TRPV1) channel to accommodate capsazepin (a TRPV1 antagonist)^[22]^ and capsaicin (a TRPV1 agonist),^[23]^ respectively.

There are also two known antagonistic sites. The first site was determined to accommodate A‐967079^[14]^ and GDC‐0334 (a highly potent, selective, and orally bioavailable TRPA1 antagonist),^[24]^ and it was observed to overlap with the first agonistic pocket (TM5‐PH1‐TM6) that binds menthol, anethole and general anesthetics (Figure 1). The second site is located in the linker domain (TM4–TM5), above the TRP‐like domain in C‐terminal and many antagonists have been shown to interact with that site. In that pocket, the antagonist HC‐030031 (Figure 2) has been suggested to form a stable hydrogen bond (H‐bond) with Asn855 (in TM4–TM5 linker).^[25]^ Another xanthine derivative antagonist, ‘compound (cpd) 3–60’, structurally similar to HC‐030031 (Figure 2), has been shown by cryogenic electron microscopy (cryo‐EM) to interact with that site in hTRPA1 through hydrogen bonds formed with Glu854 (in TM4–TM5 linker) and Asn855, as well as with Trp711 (in pre‐TM1 region) (Protein Data Bank [PDB] structure IDs: 7OR0^[26]^ and 7OR1^[27]^). Additionally, this compound can engage in π–π interactions with Trp711 and Phe853 (in TM4–TM5 linker). Furthermore, at the same antagonist site a tetrahydrofuran‐based antagonist (‘cpd 21’) complexed with a closed‐state hTRPA1 in a cryo‐EM structure (PDB ID:7JUP^[28]^) forms hydrogen bonds with His983, Gln979 (in TRP‐like domain) and Arg852 (in TM4–TM5 linker) and π–π interactions with Trp711 (Figures 1 & 2).

In the last decade, stilbenoids have been screened for their biological activity at TRPA1 (see Supporting Information, Table S2). In 2013, Yu et al.^[6]^ studied the effect of stilbenoids on TRP channels using *in vitro* and *in vivo* studies. They showed that resveratrol can reverse the effect of AITC through a non‐competitive mechanism, as well as suppress the activity of 2‐aminoethoxy diphenyl borate, a non‐electrophilic TRPA1 agonist, in HEK293 cells expressing mTRPA1. On the other hand, resveratrol did not exhibit the same effect against the TRPV1 agonist capsaicin in rTRPV1. Furthermore, pretreatment with resveratrol suppressed AITC activity in rat dorsal root ganglion (DRG) sensory neurons where TRPA1 is co‐expressed with TRPV1. Unlike resveratrol, pinosylvin monomethyl ether (PME) competitively inhibited rTRPV1 activation by capsaicin in HEK293 cells and rat DRG neurons. However, *trans*‐stilbene (*E*‐1,2‐diphenylethylene) (Figure 2), also showed no inhibitory effect on either mTRPA1 or rTRPV1, emphasizing the importance of the phenolic groups for the biological activity of stilbenoids. Furthermore, pretreatment with resveratrol or PME was found to reduce acute nocifensive behaviors in rats induced by AITC or capsaicin, respectively. In sum, the study demonstrated that resveratrol and PME inhibit the activation of TRPA1 and TRPV1 channels, respectively, but it remained unclear if the stilbenoids do that by direct modulation of the ion channel or somehow indirectly.^[6]^

In 2015, Moilanen and co‐workers illustrated a dose‐dependent suppressive effect of pinosylvin and resveratrol on AITC‐induced Ca^2+^ influx in HEK293 cells expressing hTRPA1 using a Fluo‐3‐AM assay. This was further confirmed by patch clamp experiments. Interestingly, at the highest concentration (100 μM), resveratrol and pinosylvin also activated the hTRPA1 channel to some extent. Moreover, these compounds eliminated AITC‐induced edema in mouse.^[29]^

In 2016, Nalli and coworkers reported that resveratrol and pinosylvin act as ‘true’ antagonists of rTRPA1. Unlike the other stilbenoid analogs they studied, resveratrol and pinosylvin did not activate rTRPA1 in transfected HEK293 cells, as determined by a Fluo‐4 assay, but they inhibited the effect of AITC through a non‐desensitization mechanism. On the other hand, PME and the other studied stilbenoid analogues displayed an inducing effect on rTRPA1 when used alone while showing a suppressive effect in the presence of AITC. It was also observed that none of the studied stilbenoids could significantly modulate hTRPV1 channel.^[6]^ However, in line with Yu's study,^[6]^ PME acted as a modest hTRPV1 inhibitor against capsaicin. So far, PME has been considered a dual inhibitor of hTRPV1 and rTRPA1.^[30]^

In 2017, Nakao et al. demonstrated that resveratrol, pinosylvin and PME evoked calcium influx in hTRPA1‐expressing HEK293 cells using a Fluo‐4 assay, although their potency was not as pronounced as that of AITC. However, only resveratrol and PME were able to inhibit the effect of AITC through a desensitization mechanism. Interestingly, pinosylvin showed only agonistic activity in this study, which contrasts with the previous research findings.^[31]^ This study raises the question: Do all natural stilbenoids (resveratrol, pinosylvin and PME) affect TRPA1 activity through a similar mechanism or may species‐specific differences play a role in their activity on TRPA1 (human and mouse/rat)?

Despite some conflicting results in the previous studies, natural stilbenoids have primarily been identified as potential TRPA1 blockers either through a desensitization mechanism or other unknown mechanism. Based on the antagonist hypothesis, we conducted molecular docking and molecular dynamics (MD) simulation studies of five natural stilbenoids (Figure 2) to explore their putative binding site(s) at hTRPA1 and rTRPA1. We first targeted the two known binding pockets that have been identified for the TRPA1 antagonists A‐967079 and HC‐030031. Moreover, after isolating and purifying these compounds from Scot pine and Norway spruce, their biological activity against hTRPA1 (and rTRPA1 for resveratrol) was evaluated by a fluorescent imaging plate reader (FLIPR™) calcium assay in human (or rat) TRPA1‐transfected HEK293 cells.

Based on these preliminary computational and biological studies, the investigated stilbenoids did not appear to show affinity for the HC‐030031 antagonistic site (but rather the A‐967079 antagonistic site that partially overlaps with the agonistic site for menthol, anethole and general anesthetics), nor did they act as pure antagonists in the FLIPR™ assay as they also activated the channel. Therefore, a second hypothesis was explored: stilbenoids may act as TRPA1 agonists that then desensitize the channel. To investigate this, their affinity to the other agonistic, GNE‐551 binding pocket was also examined using molecular docking and MD simulations. Additionally, we used bias‐force pulling MD simulations to predict the preferred binding pocket of stilbenoids at hTRPA1.

## Results and Discussion

### Selection of the hTRPA1 Structure for Computational Studies of Stilbenoids at the A‐967079 Site

Since the first report on the full‐length hTRPA1 structure (PDB ID: 3J9P; resolution 4.24 Å^[14]^) in 2015, several cryo‐EM structures of the channel (with or without ligands) have been released (PDB IDs: 6PQO, 6PQP, 6V9V, 6V9W, 6V9X, 6V9Y, 6X2J, 6WJ5, 7JUP, 7OR0 and 7OR1) (Supporting Information, Table S3). Structural analysis of the published structures showed significant differences in the open and closed states of the selectivity filter (residue Asp915) and the lower gate (Ile957 and Val961). Most of these structures have been determined in an intermediate (partially closed) state, wherein the upper gate is open while the lower gates are closed. Only the structure complexed with iodoacetamide, an electrophilic agonist, has been successfully determined in the open state (PDB ID: 6V9X). For example, despite having been complexed with a non‐electrophilic agonist (GNE‐551), the channel is in the fully closed state (PDB ID: 6X2J).^[21]^

When we started this study, the only available hTRPA1 structure was PDB ID: 3J9P with the putative A‐967079 site approximately located in Paulsen and co‐workers’ publication^[14]^ (no ligand coordinates were provided). As the natural stilbenoids show some structural similarities with A‐967079, we assumed that they could also bind to the same site with this antagonist and performed our first docking studies at that pocket (see below). Upon the release of the other hTRPA1 structures, we examined the significance of the open/closed state of the channel on the putative pose and interactions of A‐967079 at its binding site. Therefore, A‐967079 was docked into three different hTRPA1 structures showing an intermediate, an open and a closed conformation (PDB IDs: 3J9P, 6V9X and 6X2J, respectively). These structures exhibit differences in the size of the A‐967079 binding site and the conformation of the TM domains.

**Figure 3:**
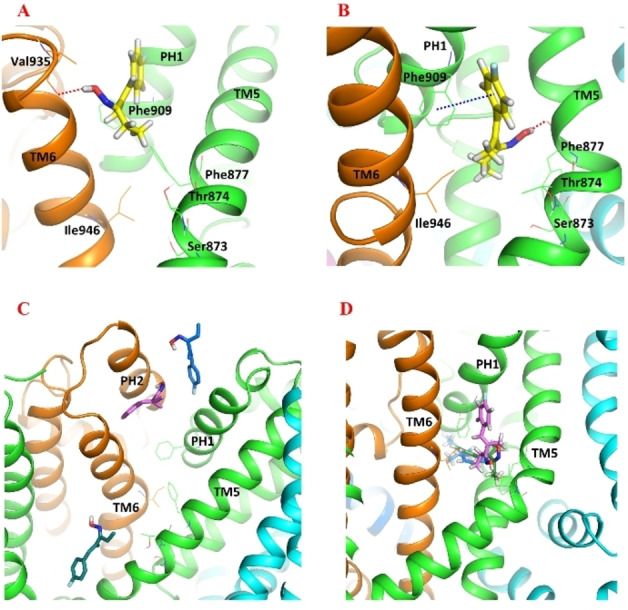
The docked pose of A‐967079 (yellow stick) in (A) the open‐state (PDB ID: 6V9X) and (B) the closed‐state (PDB ID: 6X2J) hTRPA1 structure (cartoon, chain A – orange; chain B – blue; chain C – cyan; chain D – green). The docked pose of A‐967079 (blue, pink, green sticks) after three 500‐ns parallel molecular dynamics (MD) simulations in (C) the open‐state and (D) the closed‐state hTRPA1 structure. Atom color code: nitrogen – blue; oxygen – red; fluorine – light blue; hydrogen – white. The TM domains are labelled, and the key residues are labelled (in A, B) and shown as lines. Interaction color code (dashed lines): H‐bond – red; π–π – blue. Non‐polar hydrogen atoms are omitted for clarity.

The docking results revealed that in the open‐state conformation, A‐967079 could not be positioned close enough to domain TM5 to form any interactions with the identified key residues at that site[^14^, ^32^, ^33^] (see Supporting Information, Table S1 for the residues that have been found in mutational studies to be important for the channel sensitivity to A‐967079 or for A‐967079 antagonist action). For example, A‐967079 could not reach Ser873 or Thr874 to form a hydrogen bond interaction in the open‐state structure (Figure 3A). On the other hand, although A‐967079 was successfully docked in proximity to domain TM5 in the closed state of hTRPA1, it did not adopt a pose that could form a hydrogen bond with Ser873 or Thr874, but it engaged in an aromatic interaction with Phe909 and formed a hydrogen bond to Phe877 backbone oxygen (Figure 3B). Conversely, in the intermediate‐state channel, A‐967079 docked successfully in the previously reported position at TM5‐PH1^[14]^ and was able to form a hydrogen bond with Thr874.

To rule out the possible effect of the fact that the rigid receptor docking may not always produce optimal binding poses due to non‐optimal side chain conformations at the docking site, we investigated if we could obtain a better binding pose at the open/closed state structures by performing MD simulations of the docking complexes. These 500 ns simulations were not long enough to change the open/closed state but were able to sample the local side chain conformations at the binding site. The results revealed that in the open state, A‐967079 could still not reach the TM5 domain to form any key interactions. Instead, it mostly occupied a position somewhere between the pore helices (PH1 and PH2) (Figure 3C). In the closed state, although the tail of A‐967079 could adopt different conformations, the molecule remained in close proximity to TM5 domain and even formed a hydrogen bond with Thr874 (Figure 3D). Based on these findings, it can be concluded that TRPA1 structures that are in the intermediate or closed state seem to be more optimal for studying ligand binding interactions at the A‐967079 binding site. However, as the closed state structure includes the agonist GNE‐551 that is bound at another site than A‐967079, we were convinced that we can continue using the intermediate state structure that was originally determined with A‐967079^[14]^ (although no ligand coordinates nor clear density could be found in the published structure PDB ID: 3J9P) for investigating the putative binding of stilbenoids at the A‐967079 binding pocket.

### Computational Analysis of the Natural Stilbenoids Binding at the TRPA1 Channel

**Figure 4:**
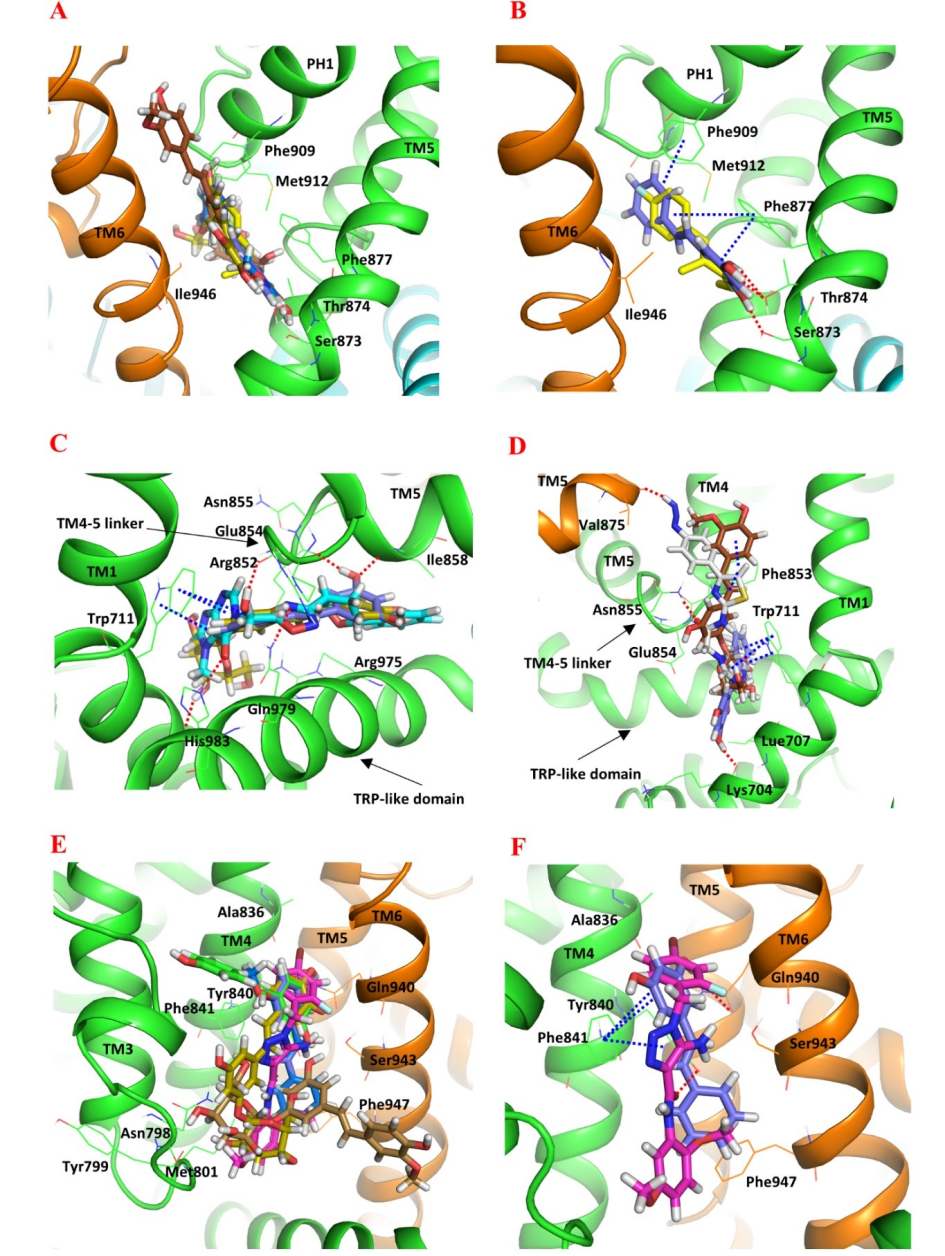
Docked poses of stilbenoids and reference compounds (sticks representation) at different ligand binding sites of hTRPA1 (cartoon representation; the two adjacent subunits are presented in green and orange). (A) The agonistic/antagonistic A‐967079 binding site of the refined intermediate‐state hTRPA1 (PDB ID: 3J9P; MD simulation frame saved at 7 ns). (B) Comparing the docked poses of pinosylvin (purple) and A‐967079 (yellow) at the A‐967079 binding site. Comparing the docked poses of the stilbenoids at the antagonistic HC‐030031 site with the reference cpd 21 (C) (PDB ID: 7JUP) and cpd 3–60 (D) (PDB ID: 7OR0). (E) The agonistic GNE‐551 binding site of the refined intermediate‐state hTRPA1. (F) Comparing the docked poses of pinosylvin and GNE‐551 (magenta) at the GNE‐551 binding site. Color of the other stilbenoids: resveratrol (green), PME (marine), astringin (olive) and isorhapontine (brown). Atom color code: nitrogen – blue; oxygen – red; fluorine – light blue; bromine – dark red; hydrogen – white. The key residues (in lines) and the TM domains are labelled. Interaction color code (dashed lines): H‐bond – red; π–π – blue.

While A‐967079 was successfully docked to its reported binding site in the intermediate‐state hTRPA1 as described above, the Glide XP GScore (−3.2 kcal/mol) and the calculated Prime/MM‐GBSA free energy of binding (−20.07 kcal/mol) for that pose suggested that the interactions at the site were not optimal (the more negative value means stronger interaction). We hypothesized that the bulky Phe877 and Phe909 at the site may easily rotate to create more space for ligand binding. Therefore, we carried out a very short (9 ns) MD simulation to identify possible hTRPA1 conformers with a more spacious A‐967079 pocket for the docking study. Overall, the potential energy of the simulation system remained stable during the MD simulation (Supporting Information, Figure S2). This short simulation was sufficient to rotate the bulky side chains at the site and, thus, to enlarge the binding site for the next docking experiments. Among the 14 snapshots taken along the 9‐ns trajectory, snapshots 9, 10, 13 and 14 had a binding cavity that had opened up due to the new side chain conformations. The snapshot 10 (at simulation time point 7 ns) with the most optimal side chain conformations making the widest binding site was selected for the docking study. The reference compound (A‐967079) along with the natural stilbenoids (resveratrol, pinosylvin, PME, astringin and isorhapontin) was then docked into the A‐967079 binding pocket located at the interface of Chain D of the selected hTRPA1 conformer. Since there are currently no good‐resolution structures of hTRPA1 in complex with A‐967079 available for comparison, we evaluated the binding poses based on the mutational data that has identified key amino acid residues important for A‐967079 activity (see Supporting Information, Table S1). All natural compounds, except isorhapontin, adopted a favorable pose where the phenolic hydroxyl groups form a hydrogen bond with Ser873 or Thr874 or both, and the aromatic structures engaged in π–π interactions with the phenyl ring of Phe877 or Phe909 or both (Figure 4A and B). Although isorhapontin exhibited a good XP GScore and a low MM‐GBSA free energy of binding (the lower the better), it could not interact with the same essential residues as the other compounds. Instead, it formed a hydrogen bond with Val948 and Pro949 from domain TM6 (Table 1).

**Table 1:** Docking results of the stilbenoids at the A‐967079 binding site of the intermediate‐state hTRPA1 (PDB ID: 3J9P, MD frame saved at 7 ns).

Compounds	XPGScore (kcal/mol)	Prime/MM‐GBSA ΔG‐bind (kcal/mol)	H‐bond^[a]^	π–π interaction
Resveratrol	−8.63	−54.97	Ser873, Thr874, Met912 (bb)	Phe877
Pinosylvin	−8.90	−58.32	Ser873, Thr874	Phe877, Phe909
PME	−8.23	−53.89	Ser873	Phe877, Phe909
Astringin	−10.87	−52.99	Thr874	Phe909
Isorhapontin	−9.88	−57.98	Val948 (bb), Pro949 (bb)	–
A‐967079	−7.53	−44.22	Thr874	Phe877

[a] Hydrogen bond formed with the polar side chain atoms of the residue if not marked with bb (backbone atom).

We also investigated the putative binding interactions of the natural stilbenoids in the antagonistic pocket of HC‐030031. Since no experimental HC‐030031‐hTRPA1 complex is available, the stilbenoids were docked into the antagonistic pocket based on the hTRPA1 cryo‐EM structures with the analogous xanthine (cpd 3–60) and hypoxanthine (cpd 21) derivatives (Figure 4C and D; Supporting Information, p. S8, Figure S3). In general, the predicted binding affinity of the natural stilbenoids for this site was weaker compared to that for the A‐967079 pocket and did not favorably compare with the predicted affinities of the reference antagonists (Supporting Information, Table S4).

Moreover, we explored the possibility that stilbenoids might interact with the other known agonistic site (GNE‐551 site) in addition to the shared agonist/antagonist site of A‐967079. We based this hypothesis on the fact that the GNE‐551 binding site in TRPA1 corresponds to the vanilloid binding site (VBS) in TRPV1 (which is shared by both agonists and antagonists^[23]^) and that the *in vitro* study by Nakao et al.^[31]^ demonstrated that stilbenoids can activate the hTRPA1 channel (and subsequently inhibit the channel through a desensitization mechanism). Based on the interactions, docking scores, and the binding free energy values, it appears that the stilbenoids show a higher affinity to the agonistic/antagonistic A‐967079 pocket at TM5‐PH1‐TM6 than the agonistic GNE‐551 pocket between TM4 of one subunit and TM5 and TM6 of the adjacent subunit (Figure 4E and F; Supporting Information, p. S11, Table S5).

### Binding of Stilbenoids to rTRPA1 vs hTRPA1

In our experimental assay, resveratrol exhibited similar activity on rTRPA1 to that observed on hTRPA1 but with lower potency (Figure 9C and D). One study indicated that the mutation of Val875 and Ile946 (in human) to glycine and methionine (in rat), respectively, resulted in complete loss of activity of piperidine carboxamides (TRPA1 agonists) on TRPA1.^[34]^ These agonistic compounds share a common binding site with A‐967079. Another study revealed that replacement of Tyr840 (in human) to phenylalanine (in rat) reduced the potency of GNE‐551 by six‐fold (see the sequence alignment of human, rat and mouse TRPA1 sequences at these binding sites in Supporting Information, Figure S4).^[21]^ Based on these studies, it could indeed be plausible that stilbenoids occupy either the A‐967079 pocket or the GNE‐551 pocket as their potency on rTRPA1 is lower. To further investigate the effect of species‐specificity on binding of resveratrol at these binding sites, a rTRPA1 model was generated. Resveratrol and the respective reference compounds were then docked to the A‐967079 and GNE‐551 pockets in the rat TRPA1 model and the results were compared with those obtained for hTRPA1. Interestingly, resveratrol's binding mode and binding free energy did not differ significantly at the A‐967079 binding site of human and rat TRPA1 (Supporting Information, Table S6). Moreover, the free energy of binding was even better for the reference compound A‐967079 at rTRPA1 than at hTRPA1 in its respective pocket (−44.22 kcal/mol at hTRPA1; −55.59 kcal/mol at the rTRPA1 model). On the other hand, resveratrol's interactions entirely mutated in the GNE‐551 pocket at the rTRPA1 model, and the free energy of binding got less negative (worsened) (from −53.00 kcal/mol at hTRPA1 to −44.85 kcal/mol at the rTRPA1 model). This worsening of the free energy of binding was observed even for GNE‐551 (from −66.83 kcal/mol at hTRPA1 to −55.68 kcal/mol at the rTRPA1 model). To further investigate the robustness of the docking results at the GNE‐551 site, three to five parallel MD simulations were run for resveratrol and GNE‐551 in the GNE‐551 pocket of h/rTRPA1.

**Figure 5:**
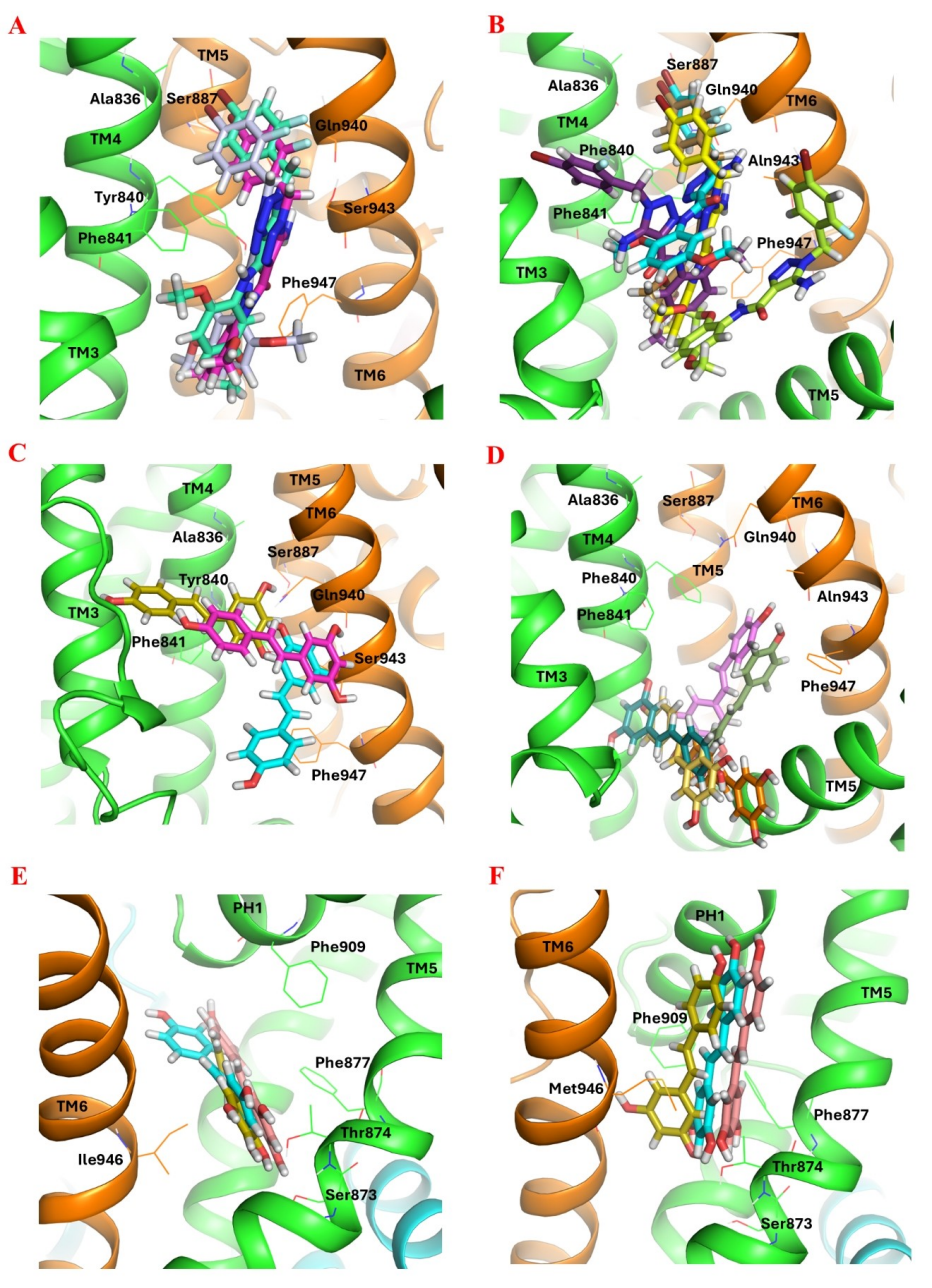
The binding pose of GNE‐551 in its binding pocket at (A) hTRPA1 (PDB ID: 6X2J; cartoon representation) and (B) the rTRPA1 model at the end of three and five 400‐ns parallel MD simulations, respectively. (C) The binding pose of resveratrol at the GNE‐551 binding site of hTRPA1 (refined intermediate state; PDB ID: 3J9P) and (D) the rTRPA1 model at the end of three and five 400‐ns parallel MD simulations, respectively. (E) The binding pose of resveratrol at the A‐967079 binding site of hTRPA1 (refined intermediate state; PDB ID: 3J9P) and (F) the rTRPA1 model at the end of three 400‐ns parallel MD simulations. The TRPA1 is represented in cartoon (subunits colored differently) and GNE‐551 and resveratrol in sticks. Atom color code: carbon – different colors based on the MD snapshot; nitrogen – blue; oxygen – red; fluorine – light blue; bromine – dark red; hydrogen – white. The key residues (in lines) and the TM domains are labelled.

The MD simulation studies illustrated that GNE‐551 in its binding pocket at hTRPA1 (PDB ID: 6X2J) was stably bound and no notable changes in binding mode or free energy of binding occurred during the 400‐ns simulations (Figure 5A and Supporting Information, Table S7, Figure S5A). However, although GNE‐551 stayed in the rTRPA1 binding pocket during the simulations, it lost the interaction with Gln940, and the binding free energy got significantly poorer in three of the five parallel simulations (Figure 5B and Supporting Information, Table S7, Figure S5B). Although resveratrol's docked pose altered slightly in the GNE‐551 pocket at hTRPA1 (refined intermediate state; PDB ID: 3 J9P), its strong hydrogen bonds with Gln940 and Ser943 kept it in the binding site and the free energy of binding remained at the same level (Figure 5C and Supporting Information, Table S7, Figure S6A). In the GNE‐551 binding pocket of the rTRPA1 model, resveratrol lost the initial interactions (Met845 [TM4], Glu864 [TM5], Phe947 [TM6]) during most of the simulations and moved out from the pocket or made new interactions with a better free energy of binding at a new site. In only one of the parallel simulations, it maintained the initial interactions and even formed an additional hydrogen bond with the alanine backbone equivalent to Ser943 in hTRPA1 (Figure 5D and Supporting Information, Table S7, Figure S6B). The interaction with Ser943 may play a crucial role in retaining resveratrol within the GNE‐551 binding pocket at hTRPA1.

To further investigate the hypothesis that stilbenoids may bind to the GNE‐551 pocket (which is seen in the reduction of activity at rTRPA1) instead of the A‐967079 pocket, another series of parallel MD simulations was conducted for resveratrol at the A‐967079 binding site of human and rat TRPA1. The results showed that resveratrol remained stable in the A‐967079 pocket at both h/rTRPA1, maintaining its binding pose and the H‐bond interactions with Thr874 or Ser873 throughout the simulations. At rTRPA1, although the initial pose of resveratrol changed at the beginning of the simulations, it eventually settled into the correct pose after 50–100 ns and even became more stable with an additional π–π interaction with Phe909. The free energy of binding did not significantly change during the 400‐ns simulations (Figure 5E and F and Supporting Information, Table S7, Figure S7A and B).

### Predicting the Binding Pocket of Stilbenoids Using Steered Molecular Dynamics Simulations

**Figure 6:**
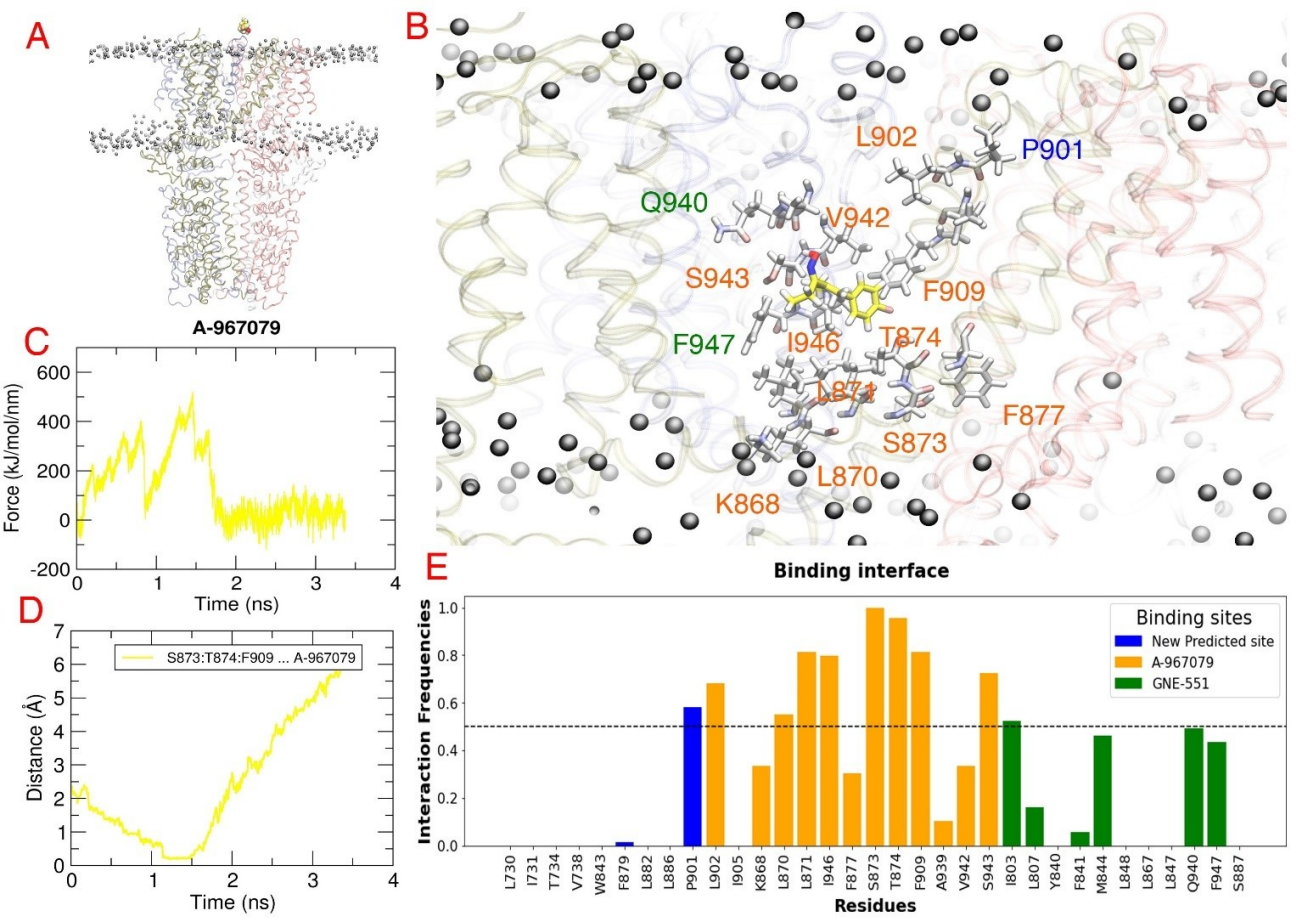
Steered molecular dynamics results for the reference compound A‐967079. (A) The SMD simulation system that consists of hTRPA1 (PDB ID: 6V9V) (ribbon representation, subunits colored differently) embedded in a POPC (1‐palmitoyl‐2‐oleoyl‐sn‐glycero‐3‐phosphocholine) membrane (black balls) and A‐967079 above the ion channel (yellow space filling model). (B) The predicted binding mode of A‐967079 (yellow sticks) obtained at the highest force. The residues forming the predicted binding pocket are presented in white sticks and labelled. (C) The force profile of A‐967079 exhibiting the force peaks during the pulling simulation. (D) Medium distance of the essential residues (S873, T874, F909) from A‐967079 indicating the time when the ligand reaches its binding pocket. (E) The contact frequency analysis of hTRPA1 residues identifies the residues that are most frequently contributing to the binding interaction with the ligand during the pulling simulation.

To further assess the affinity of pinosylvin and resveratrol for these putative binding sites, a bias‐force pulling simulation or steered molecular dynamics simulation (SMD) method was employed. It involved pulling the ligand through the TM domain of the TRPA1 channel for 3 ns. This procedure was repeated 20–25 times for each ligand and in the following paragraphs, we provide representative results of those simulations (one of the top three simulations based on the highest forces and longest time spent at the site and the most stable the interactions). Throughout this process, the bias‐force pulling simulation provided several critical insights, including a potential pathway that the ligand follows as it enters and exits the binding pocket, the profile of peak force (Fmax) and the number of interactions formed during the simulation. The method was validated using A‐967079, which entered its respective pocket after passing by Pro901 and Leu902 located in PH1. At its binding site, A‐967079 formed lipophilic interactions with residues Ile946 (TM6), Leu870, Leu871 (TM5) and Phe909 (PH1), as well as polar interactions with Ser873 or Thr874 (TM5). The time range required for A‐967079 to enter the pocket was between 0.86 and 1.15 ns, with the Fmax peak calculated as 518 kJ/mol, occurring at 1.459 ns (Figure 6).

**Figure 7:**
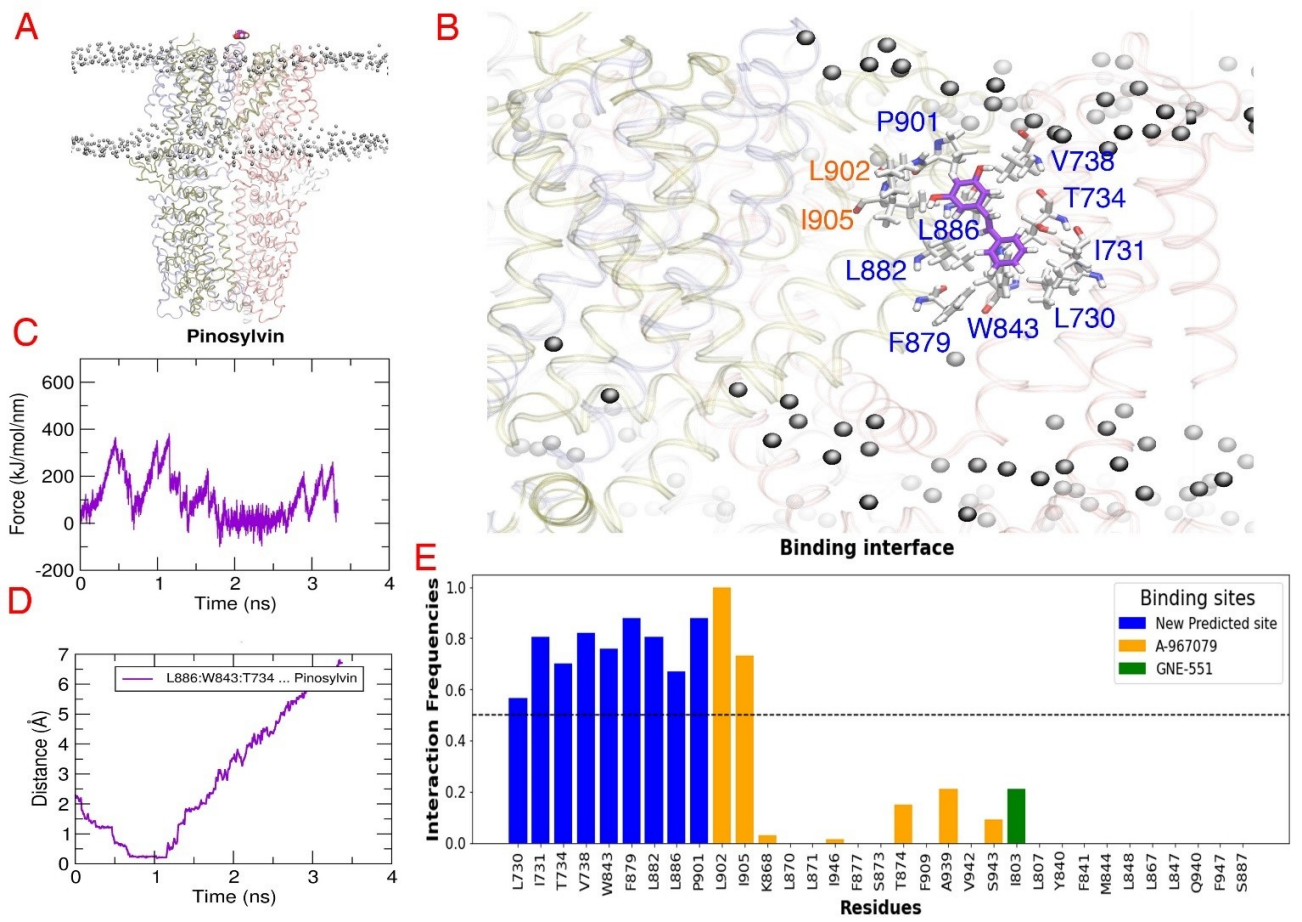
Steered molecular dynamics (SMD) results for the natural stilbenoid pinosylvin. (A) The SMD simulation system that consists of hTRPA1 (PDB ID: 6V9V) (ribbon representation, subunits colored differently) embedded in a POPC (1‐palmitoyl‐2‐oleoyl‐sn‐glycero‐3‐phosphocholine) membrane (black balls) and pinosylvin above the ion channel (purple space filling model). (B) The predicted binding mode of pinosylvin (purple sticks) obtained at the highest force. The residues forming the predicted binding pocket are presented in white sticks and labelled. (C) The force profile of pinosylvin exhibiting the force peaks during the pulling simulation. (D) Medium distance of the most contacting residues (L886, W843, T734) from pinosylvin indicating the time when the ligand reaches the predicted binding pocket. (E) The contact frequency analysis of hTRPA1 residues identifies the residues that are most frequently contributing to the binding interaction with the ligand during the pulling simulation.

Interestingly, pinosylvin rather occupied a new predicted pocket between 0.80 and 1.15 ns with the Fmax peak measured at 381 kJ/mol. This pocket was located at the interface of TM1 and TM4 domains from one subunit and involved TM5 domain from the other subunit. Pinosylvin primarily engaged in lipophilic interactions with residues Leu730, Ile731 and Val738 from TM1, and Trp843 from TM4 domain, as well as Phe879, Leu882 and Leu886 from TM5. Additionally, a potential hydrogen bond was observed with Thr734 (TM1) (Figure 7).

**Figure 8:**
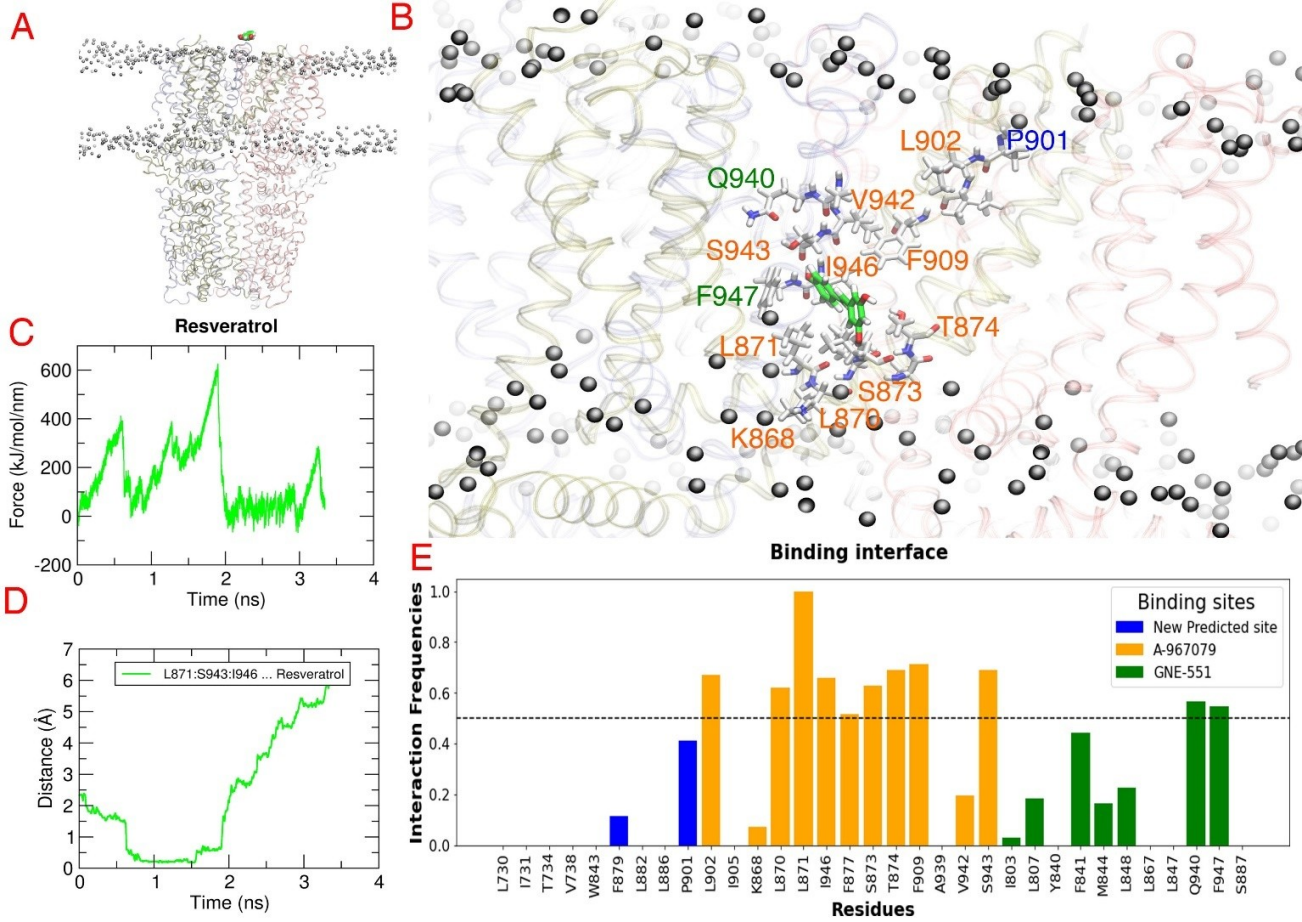
Steered molecular dynamics (SMD) results for the natural stilbenoid resveratrol. (A) The SMD simulation system that consists of hTRPA1 (PDB ID: 6V9V) (ribbon representation, subunits colored differently) embedded in a POPC (1‐palmitoyl‐2‐oleoyl‐*sn*‐glycero‐3‐phosphocholine) membrane (black balls) and resveratrol above the ion channel (green space filling model). (B) The predicted binding mode of resveratrol (green sticks) obtained at the highest force. The residues forming the predicted binding pocket are presented in white sticks and labelled. (C) The force profile of resveratrol exhibiting the force peaks during the pulling simulation. (D) Medium distance of the most contacting residues (L871, S943, I946) from resveratrol indicating the time when the ligand reaches the predicted binding pocket. (E) The contact frequency analysis of hTRPA1 residues identifies the residues that are most frequently contributing to the binding interaction with the ligand during the pulling simulation.

On the other hand, resveratrol demonstrated affinity to the A‐967079 binding site, interacting with Leu871, as well as Ile946 and Ser943 (TM6 of adjacent subunit). In some simulations (2 out of 3), there was a potential hydrogen bond with Thr874. Resveratrol remained in the pocket between 1.11 and 1.28 ns, with the Fmax peak measured at 389 kJ/mol (Figure 8). Similar to pinosylvin, resveratrol also resided in the new predicted pocket and engaged in both lipophilic and polar interactions, although the recorded Fmax peak (286 kJ/mol) in this pocket was lower than that in the A‐967079 pocket. Interestingly, neither resveratrol nor pinosylvin showed any affinity to the GNE‐551 binding site. Taken together, while the bias‐force pulling simulations failed to confirm the affinity of resveratrol and pinosylvin for the GNE‐551 binding pocket, they suggested a novel binding site for pinosylvin.

### Biological Activity of the Natural Stilbenoids on hTRPA1

**Figure 9:**
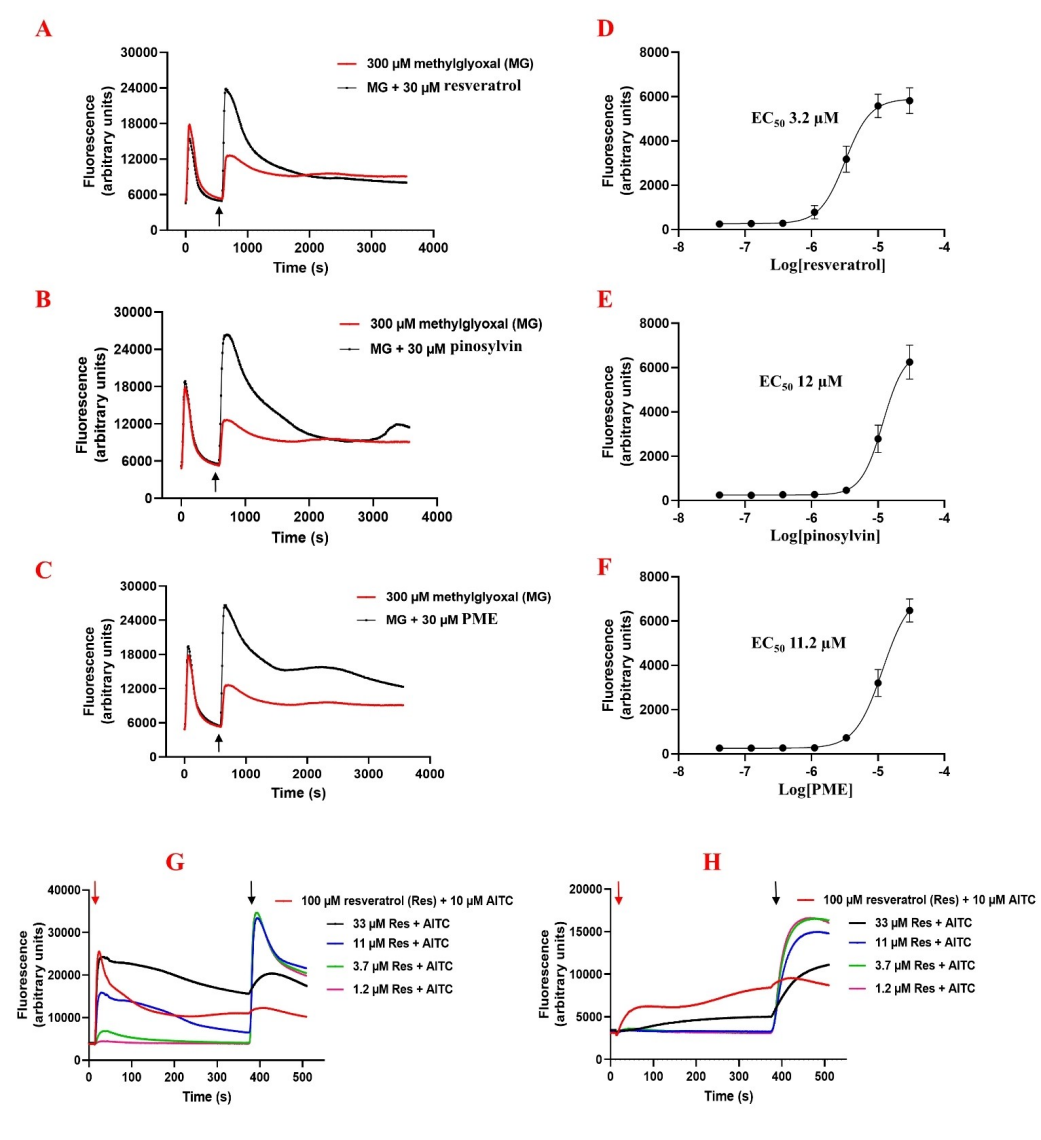
The FLIPR™ assay shows agonistic activity of resveratrol (A), pinosylvin (B) and PME (C) in the presence of methylglyoxal (MG) in human TRPA1‐induced HEK293 cells. The stilbenoids were applied at the 10 minute time point (marked by the black arrow). The curves correspond to relative fluorescent units, reflecting the intracellular Ca^2+^ concentration. The EC_50_ curve of resveratrol (D), pinosylvin (E) and PME (F), data are means of five separate determinations. Effects of resveratrol on (G) hTRPA1 and (H) rTRPA1 in the Ca^2+^ assay. Resveratrol was added at different concentrations (marked by the red arrow) followed by the addition of AITC (10 μM) dispensed on cells after 6 minutes (marked by black arrow).

Natural stilbenoids were tested on TRPA1‐transfected HEK293 by using the FLIPR™ assay. Surprisingly, none of the compounds could inhibit the Ca^2+^ response evoked by methylglyoxal (MG). Furthermore, it seemed that resveratrol, pinosylvin and PME could even boost the second phase of methylglyoxal activation, indicative of TRPA1 agonist activity (Figure 9A–C). Hence, their agonistic activity on hTRPA1 was examined in comparison with AITC. However, their activity was not as intense as that of AITC. Resveratrol, pinosylvin and PME activated the channel with EC_50_ of 3.2±0.2 μM, 12.0±1.1 μM and 11.2±0.8 μM, respectively (Figure 9D–F).

Unlike the studies by Yu et al.^[6]^ and Nalli et al.,^[30]^ which indicated that resveratrol and pinosylvin act as pure TRPA1 antagonists, our study suggests that natural stilbenoids, except for their glycosides, can act as moderate TRPA1 agonists rather than pure antagonists. To understand if the stilbenoids can inhibit TRPA1 through channel desensitization, as shown in the study by Nakao et al.^[31]^ and to investigate the role of species‐specific differences in their activity, resveratrol in different concentrations was applied 6 minutes before AITC (10 μM) on human and rat TRPA1. As expected, resveratrol could significantly counteract the effect of AITC at higher concentrations (at 33 and 100 μM) and in a dose‐dependent manner in both species. However, resveratrol evoked a more intense response on hTRPA1 than the rTRPA1 channel. Resveratrol at 100 μM, could reach 35 % and 70 % of the maximal response (10 μM AITC) on rTRPA1 and hTRPA1, respectively (Figure 9G and H). It can be concluded that stilbenoids cause TRPA1 channel desensitization, which is then detected as antagonism in certain type of measurements (when cells are preincubated with the compound).

Although astringin and isorhapontin showed acceptable values in the molecular docking studies and interacted with the channel in the known binding sites through their hydroxyl groups, they were entirely inactive in the *in vitro* study. They have higher hydrophilicity compared to the stilbenoid aglycons that decreases their membrane penetration and likely prevents them from accessing the possible binding pockets. A previous study showed that a part of piceid (resveratrol's glucoside) can be absorbed in an enterocyte via SGLT1 (sodium‐dependent glucose transporter 1) and a notable amount can be metabolized into resveratrol in the intestinal lumen by LPH (lactase‐phlorizin hydrolase) and CBG (cytosolic‐ß‐glucosidase) enzymes.^[35]^ So, an *in vivo* study is needed to confirm the stilbenoid glucoside's (or their metabolites’) effect on TRPA1. In line with our biological study, the stilbenoids were predicted to have the lowest affinity at the pure antagonist site (the HC‐030031 binding pocket) based on the docking studies. In addition, their predicted binding free energy at the shared agonistic/antagonistic (A‐967079 and menthol/anesthetics binding pocket) is comparable to or even better than that at the fully agonistic (GNE‐551) binding site. Considering that stilbenoids (aglycons) inhibit the channel with a similar mechanism as anesthetics do and have a similar size as propofol, anethole and A‐967079, they may well share the same binding site with these compounds. However, a significant reduction in the activity of resveratrol on rTRPA1 made us consider the possibility that stilbenoids may (also) occupy the GNE‐551 binding pocket. Our docking and simulation studies suggest that the binding affinity of resveratrol and GNE‐551 to the GNE‐551 binding pocket of rTRPA1 is poorer than their affinity to that pocket in hTRPA1. Finally, despite all these *in silico* binding site/mode investigations and biological studies, unveiling the exact binding pocket and binding mode of stilbenoids at hTRPA1 needs further experimental studies.

## Conclusions

While the activity of stilbenoids on TRPA1 has been evaluated in several studies using different methods, their binding site at TRPA1 remains unexplored. Natural stilbenoids and their analogs have demonstrated a moderate effect on TRPA1, which renders them promising templates for designing novel anti‐inflammatory or analgesic compounds. Therefore, identifying the binding site and favorable interactions of natural stilbenoids at TRPA1 is crucial for facilitating the rational design and development of more potent and possibly selective therapeutic compounds based on their structure. Therefore, by using molecular docking and MD simulations, coupled with *in vitro* studies, we aimed at predicting the putative binding pocket of stilbenoids in this channel.

Our *in vitro* study was in line with some previous findings, suggesting that the stilbenoids act as TRPA1 channel agonists, likely inhibiting the channel through a desensitization mechanism. Our docking and MD simulation analysis further supported this result by predicting that stilbenoids exhibit higher affinity to the known agonistic sites than the antagonistic site.

While our initial molecular docking study indicated a slightly stronger binding free energy of stilbenoids at the A‐967079 binding site, the experimental comparison of resveratrol's activity on rat and human TRPA1 in the light of previous mutational studies suggested that stilbenoids may occupy the same agonistic pocket as GNE‐551. Subsequent molecular docking and MD simulation studies of resveratrol at both the GNE‐551 and A‐967079 binding sites of rat and human TRPA1 supported the findings of the biological assay, with a preference for the GNE‐551 site. Although the bias‐force pulling simulations could not confirm the affinity of resveratrol and pinosylvin to the GNE‐551 binding pocket, they predicted a completely new binding pocket for pinosylvin at the interface of TM1 and TM4 domains from one subunit and TM5 domain from the adjacent subunit.

However, determination of the exact binding site of stilbenoids remains elusive and may require further phylogenetic and mutational studies. Alternatively, structural determination of TRPA1 in complex with pinosylvin could offer a more definitive approach to resolving this matter in the future.

## Experimental Section

### Computational Studies

In general, molecular modeling and visualization of structures was carried out using Schrödinger's Maestro Molecular Modeling Suite (Releases 2020‐2, 2021‐1, 2021‐3, 2023‐2) and the PyMOL Molecular Graphics System (versions 2.5.5, 3.0) (Schrödinger, LLC, New York, NY).

**Preparation of protein structures**. The cryo‐EM structures of hTRPA1 (PDB IDs: 3J9P, 6X2J, 6V9X, 6V9V, 7JUP, 7OR0) were retrieved from the Protein Data Bank (PDB; www.rcsb.org).^[36]^ The rTRPA1 model (see Comparative modeling of rat TRPA1 section) and hTRPA1 structures were prepared using the Protein Preparation Wizard of Maestro^[37]^ with default parameters; all hydrogens were added and optimized at pH 7.0 after which the whole structure was shortly energy‐minimized in the OPLS3,^[38]^ OPLS3e^[39]^ and OPLS4^[40]^ force field (heavy atoms were constrained to root‐mean‐square deviation of maximum 0.3 Å).

**Refinement of the A‐967079 binding site in the intermediate‐state hTRPA1**. Since the putative TRPA1 antagonist A‐967079 binding site that was observed by Paulsen et al.^[14]^ is relatively tight in their published cryo‐EM structure of the hTRPA1 ion channel (PDB ID: 3J9P; resolution 4.24 Å), docking the antagonist (or the stilbenoids) directly into that pocket in this structure was not possible. Visual inspection of the binding site region with the PyMOL visualization software (v. 1.8.6.1) revealed that for example the bulky residue Phe877 makes the binding site narrow but other side chain conformations of that residue could open the pocket for small molecules. Thus, we wanted to see if a subtle structural refinement by a short MD simulation would be enough to generate side chain conformations that open the A‐967079 pocket for the docking studies.

The hTRPA1 structure (PDB ID: 3J9P) was prepared with the Protein Preparation Wizard^[37]^ of Maestro (v. 11.0.015). The ion channel structure was then simulated in an explicit solvent box of TIP3P^[41]^ water with Cl^−^ as neutralizing counter ions, using the AMBER ff03 force field^[42]^ and the AMBER16 MD simulation software.^[43]^ After minimizing and equilibrating (for 500 ps) the solvated system as described previously,^[44]^ the production simulation was run at 300 K and at 1 bar pressure for ca. 9 ns. VMD^[45]^ v. 1.9.2 was used to take snapshots at every 600 ps of the simulation trajectory, starting at 1 ns. The snapshot structures were then visually analyzed in PyMOL.

**Comparative modeling of rat TRPA1**. The rTRPA1 sequence was obtained from the UniProt Knowledgebase (UniProtKB: Q6RI86). A BLAST (Basic Local Alignment Search Tool)^[46]^ search for the sequence was run to find a suitable template in the Protein Data Bank (PDB)^[47]^ for comparative (homology) modeling of its three‐dimensional (3D) structure. Among protein structures with similar E‐values (0.0) and Sequence Identity (79.96 %), the hTRPA1 cryo‐EM structure (PDB ID: 6V9V)^[48]^ was selected due to the highest resolution (2.60 Å). The 3D structure model of rTRPA1 was generated using Modeller (v. 9.24).^[49]^ The modelling alignment was created with Clustal Omega^[50]^ and manually curated. Out of the 20 generated alternative models, the one with the best Discrete Optimized Protein Energy (DOPE) score^[51]^ was selected. The model was further evaluated by superimposing it on the template structure using PyMOL (version 2.5.5) and the stereochemical quality of the model was verified with MolProbity.^[52]^

**Preparation of ligands**. The 2D structures of the natural stilbenoids (Figure 2) and the reference molecules (A‐967079 and HC‐030031) were retrieved from ChemSpider database (www.chemspider.com). The structure of GNE‐551 was obtained from cryo‐EM structure (PDB ID: 6X2J) and prepared using the LigPrep module of Maestro (Schrödinger Releases 2020‐2: LigPrep, Schrödinger, LLC, New York, NY, 2020) software. Possible protonation states were generated using the OPLS3 or OPLS3e force field at pH 7±2.

**Molecular docking**. Molecular docking was conducted with the Glide module of Maestro.[^53^, ^54^, ^55^] The docking site was defined with the Receptor Grid Generation tool of Maestro. In the intermediate‐state hTRPA1 structure (PDB ID: 3J9P), the enclosing cubic grid was placed at the centroid of Ser873, Thr874, Phe877 and Phe909 residues for the A‐967079 binding site,^[14]^ and Aln836, Tyr840, Phe841 of one chain and Ser887, Gln940, Phe947 of the adjacent chain for the GNE‐551 binding site.^[21]^ The experimental ligand poses of GNE‐551 (PDB ID: 6X2J) and cpd 21/cpd 3–60 (PDB IDs: 7JUP/7OR0) were used to generate the docking grid for redocking validation and for docking to the HC‐030031 subsites, respectively. Ligand diameter midpoint box was set to 10 Å×10 Å×10 Å. The length limit for ligands to be docked was set to 15 Å. The extra‐precision (XP) mode of docking was used docking and five poses per ligand were written out at most. The compound poses were ranked based on the XP GScore and the interactions they made with the essential binding site residues.

**Binding free energy calculation**. The top poses of individual ligands ranked by Glide XP score were selected for the binding free energy calculations with the Prime/MM‐GBSA module of Maestro.^[56]^ The approximate energies were calculated using the VSGB 2.0 solvation model,^[57]^ OPLS3, OPLS3e or OPLS4 force field and allowing the residues within 5 Å from the ligand to move. Sampling of the flexible residues was done by minimization.

**Conventional Molecular Dynamics simulations**. The ligand‐TRPA1 simulation systems were generated with the System Builder tool of the Desmond module as implemented in Maestro (Schrödinger Release 2020‐2: Desmond Molecular Dynamics System, D. E. Shaw Research, New York, NY, USA, 2020. Maestro‐Desmond Interoperability Tools, Schrödinger, New York, NY, USA, 2020).^[58]^ The systems consisted of the POPC (1‐palmitoyl‐2‐oleoyl‐*sn*‐glycero‐3‐phosphocholine) membrane embedded TRPA1 (PDB IDs: 6X2J, 6V9X and the refined 3J9P, and the rTRPA1 model) and the docked or experimental poses of the ligands (A‐967079, GNE‐551, resveratrol) in an orthorhombic box (size: 10 Å×10 Å×10 Å) filled with explicit TIP3P water.^[59]^ Periodic boundary conditions and the OPLS4 force field were applied. The systems were neutralized by adding Cl‐ counter ions and the salt (NaCl) concentration was set to 150 μM. A cut‐off radius of 9 Å was used for short‐range interactions and the long‐range electrostatics were handled with the U‐series method.^[60]^ After the system relaxation, parallel production simulations were run for 400 or 500 ns at constant temperature (300 K) and pressure (1.01325 bar) using randomized seed velocities according to our previously reported simulation protocol.^[61]^

**Steered Molecular Dynamics simulations**. The simulation system of hTRPA1 (PDB ID: 6V9V) embedded in a POPC membrane was constructed utilizing the Charmm‐GUI web‐based platform^[62]^ (https://www.charmm‐gui.org/). The membrane consisted of a POPC‐only lipid model (126×125 in the upper and lower leaflets), which was employed to fully enclose the TM domain of TRPA1. This was achieved using the fully automated “membrane‐bilayer builder“^[63]^ panel with the CHARMM36m force field.^[64]^

The parameters for pinosylvin, resveratrol, and A‐967079 were generated using the “Ligand Reader and Modeller” of the Charmm‐GUI platform.^[65]^ The ligands were positioned in the extracellular area above the pore using VMD (version 1.9.3). The Cα atoms of the TRPA1 channel were restrained to a fixed position, while the center‐of‐mass of the entire ligands was assigned to an external bias‐potential (constant velocity (v)=0.003 nm ps^−1^ and spring constant (k)=450 kJ mol^−1^ nm^−2^). Additionally, a range of external forces (v=0.05, 0.02, 0.01, 0.009, 0.005, 0.004, 0.003, 0.002 and 0.001 and k=1000, 800, 500, 450, 400, 350, 300 and 200) were also tested on the ligands to identify the plausible force that enables the ligands to pass through the binding site residues of TRPA1 along the Z axis (i.e perpendicular to the membrane plane). Subsequently, all systems underwent a 3 ns×20–25 replicates pulling simulation for each ligand, and output frames were recorded at every 1 ps interval. The trajectory outputs were then utilized to estimate the total force (F) required to transit the ligand through TRPA1.

The MD trajectories were visually represented using VMD. The output from the simulation trajectories was then plotted using the GRACE software (http://plasma‐gate.weizmann.ac.il/Grace/).

### Isolation of Natural Stilbenoids

Pinosylvin and PME were isolated from a blend of Norway spruce and Scot pine knotwood through sequential extractions with polar and nonpolar solvents, followed by purification via column chromatography.^[66]^ Stilbene glucosides, astringin and isorhapontin, were extracted from the fresh inner bark of Norway spruce by acetone and further purified by column chromatography to achieve a purity of over 95 %. The purity of the isolated stilbenoids was evaluated using GC‐MS. This in‐house method and the results were described in detail in our published article.^[67]^

### Biological Assay

To determine the antagonism of the test compounds, the endogenous TRPA1 agonist methylglyoxal was used as channel activator and measurements were performed using fluorometric imaging plate reader FLIPR^tetra^ (Molecular Devices). Methylglyoxal has been shown to evoke a biphasic activation of the TRPA1 ion channel when changes in intracellular calcium concentration is measured.

One day prior the experiments, human or rat TRPA1‐inducible HEK293 cells were plated onto 384‐well CellBind, clear‐bottom black walled plates (Corning) at a density of 12 000 cells/well in medium supplemented with 1 mM isopropyl β‐D‐1‐thiogalactopyranoside (IPTG) to induce the TRPA1 expression. Detailed cell culture description can be found in an earlier publication.^[68]^

On the experiment day, after removing the growth medium, the cells were loaded with Calcium 6 Assay reagent and incubated for 90 min at 37 °C in the dark.

For the antagonism studies, first 300 μM methylglyoxal was added by the plate reader to activate the hTRPA1 expressing cells. Concentration series (0.04–30 μM) of the test compounds were dispensed by FLIPR^tetra^ 10 min after methylglyoxal addition with the measurement continuing for 50 min. Inhibition of the second, sustained activation of the receptor was analyzed.

In case when the effect of methylglyoxal was clearly boosted by compound application, agonism on hTRPA1 was confirmed and EC_50_ value assessed in experiments where just compound dilutions were dispensed on the cells by FLIPR^tetra^ and the signal was detected for 120 seconds. Similar experiments were performed also using HEK293 cells which do not express TRPA1 to verify that the calcium influx evoked by the compounds is mediated through TRPA1 channels (Supporting Information, Figure S8).

In addition, resveratrol was studied using the following protocol: Concentration series (0.05–100 μM) of resveratrol was dispensed on both human and rat TRPA1 expressing cells and acute agonism was measured. After 6 minutes of resveratrol addition, a known TRPA1 activator, 10 μM AITC, was applied on cells and the effects were measured for 120 seconds.

Test compounds were dissolved as 10 mM DMSO stock solutions, and the dilution series were prepared in Probenecid Ringer.^[68]^ All the experiments were performed at 37 °C, the excitation wavelength was 470–495 nm and emission was measured at 515–575 nm.

In EC_50_ calculations, the fluorescence values were normalized by subtracting the baseline value from the maximum value measured for each well. EC_50_ values were determined from dose‐response curves using nonlinear regression curve fit with variable slope model by GraphPad Prism version 8.0.2. Dose‐response curves were constructed from a mean of 5 different assay plates having 7 compound concentrations and 4 separate wells at each condition. The results are presented as EC_50_±SEM.

## Conflict of Interests

The authors declare no conflict of interest.

1

## Supporting information

As a service to our authors and readers, this journal provides supporting information supplied by the authors. Such materials are peer reviewed and may be re‐organized for online delivery, but are not copy‐edited or typeset. Technical support issues arising from supporting information (other than missing files) should be addressed to the authors.

Supporting Information

## Data Availability

The data that support the findings of this study are available in the supplementary material of this article.
