# Supplementary material for: Insights into Molecular Interactions and Biological Effect of Natural Stilbenoids at the TRPA1 Ion Channel
Source: ChemMedChem. 2024 Nov 20;20(3):e202400501. doi: 10.1002/cmdc.202400501 (PMC11793850; doi:10.1002/cmdc.202400501)
Supplement: Supplementary file 1 — Supporting Information [file CMDC-20-e202400501-s001.pdf]

ChemMedChem

Supporting Information

Insights into Molecular Interactions and Biological Effect of Natural Stilbenoids at the TRPA1 Ion Channel

Atefeh Saadabadi,* Marja Rantanen, Parthiban Marimuthu, Ari-Pekka Koivisto, Patrik C. Eklund, and Outi M. H. Salo-Ahen*

Insights into Molecular Interactions and Biological Effect of Natural Stilbenoids at The TRPA1 Ion Channel

Atefeh Saadabadi,*[a,b,c] Marja Rantanen,[d] Parthiban Marimuthu,[a,c,e] Ari-Pekka Koivisto,[d] Patrik C. Eklund,[b] Outi M. H. Salo-Ahen*[a,c]

[a] A. Saadabadi, P. Marimuthu, O. M. H. Salo-Ahen
Pharmaceutical Sciences Laboratory, Faculty of Science and Engineering
Åbo Akademi University,
Tykistökatu 6, 20520 Turku, Finland.
Email: outi.salo-ahen@abo.fi; atefeh.saadabadi@abo.fi
[b] A. Saadabadi, P. C. Eklund
Laboratory of Molecular Science and Engineering, Faculty of Science and Engineering
Åbo Akademi University
Henrikinkatu 2, 20500 Turku, Finland
[c] A. Saadabadi, P. Marimuthu, O. M. H. Salo-Ahen
Structural Bioinformatics Laboratory, Faculty of Science and Engineering
Åbo Akademi University
Tykistökatu 6, 20520 Turku, Finland
[d] M. Rantanen, A. -P. Koivisto
Pain Therapy Area, R&D
Orion Pharma
Tengströminkatu 8, 20360 Turku, Finland
[e] P. Marimuthu
Center for Global Health Research, Saveetha Medical College
Saveetha Institute of Medical and Technical Sciences
Chennai-602 105, India

Table of contents:

**Figure S1**	Structures of electrophilic and non-electrophilic TRPA1 modulators.	p. S3
**Table S1**	Reported binding sites of electrophilic and non-electrophilic TRPA1 modulators	p. S4
**Table S2**	Summary of the effect of stilbenoids on TRPA1	p. S5
**Table S3**	The cryo-EM structures of TRPA1 in the Protein Data Bank (PDB)	p. S6
**Figure S2**	Potential energy of the molecular dynamics (MD) simulation system during the equilibration (500 ps) and the consequent 9-ns production simulation of human TRPA1 (PDB ID: 3J9P) using the Amber 16 MD simulation package.	p. S7
**Text**	The stilbenoids in the HC-030031 binding pocket of hTRPA1	p. S8
**Figure S3**	Experimental binding poses of cpd 21 and cpd 3-60 in the HC-030031 pocket at their respective subsites of hTRPA1 (PDB IDs: 7JUP and 7OR0 superimposed) and the docked pose of HC-030031 compared to the experimental pose of cpd 3-60 (PDB ID: 7OR0) and cpd 21 (PDB ID: 7JUP).	p. S9
**Table S4**	Docking results of the stilbenoids at the antagonistic HC-030031 binding site of hTRPA1 (PDB ID: 7JUP; 7OR0)	p. S10
**Text**	The stilbenoids in GNE-551 binding pocket of hTRPA1	p. S11
**Table S5**	Docking results of the stilbenoids at the agonistic GNE-551 binding site of the intermediate-state hTRPA1 (PDB ID: 3J9P, MD frame saved at 7 ns)	p. S11
**Table S6**	Docking results of resveratrol at the A-967079 (antagonist) and GNE-551 (agonist) binding sites of intermediate-state hTRPA1 (PDB ID: 3J9P, MD frame saved at 7 ns) and the rTRPA1 model	p. S12
**Table S7**	Binding free energy of resveratrol at the A-967079 (antagonist) and GNE-551 (agonist) binding sites of intermediate-state hTRPA1 (PDB ID: 3J9P, MD frame saved at 7 ns) and the rTRPA1 model at the end of 400-ns parallel MD simulations.	p. S13
**Figure S4**	Sequence alignment of human, rat and mouse TRPA1 for A-967079 and GNE-551 binding sites	p. S14
**Figure S5**	Ligand RMSD plots of GNE-551 in its binding site at hTRPA1 (PDB ID: 6X2J) and the rTRPA1 model during repeated (parallel) 400-ns molecular dynamics (MD) simulations	p. S15
**Figure S6**	Ligand RMSD plots of resveratrol in the GNE-551 pocket at hTRPA1 (refined intermediate state; PDB ID: 3J9P) and the rTRPA1 model during repeated (parallel) 400-ns molecular dynamics (MD) simulations.	p. S16
**Figure S7**	Ligand RMSD plots of resveratrol in the A-967079 pocket at hTRPA1 (refined intermediate state; PDB ID: 3J9P) and the rTRPA1 model during repeated (parallel) 400-ns molecular dynamics (MD) simulations.	p. S17
**Figure S8**	The FLIPR™ assay shows activity of pinosylvin (A), PME (B) and resveratrol (C) in non-transfected HEK293 cells	p. S18

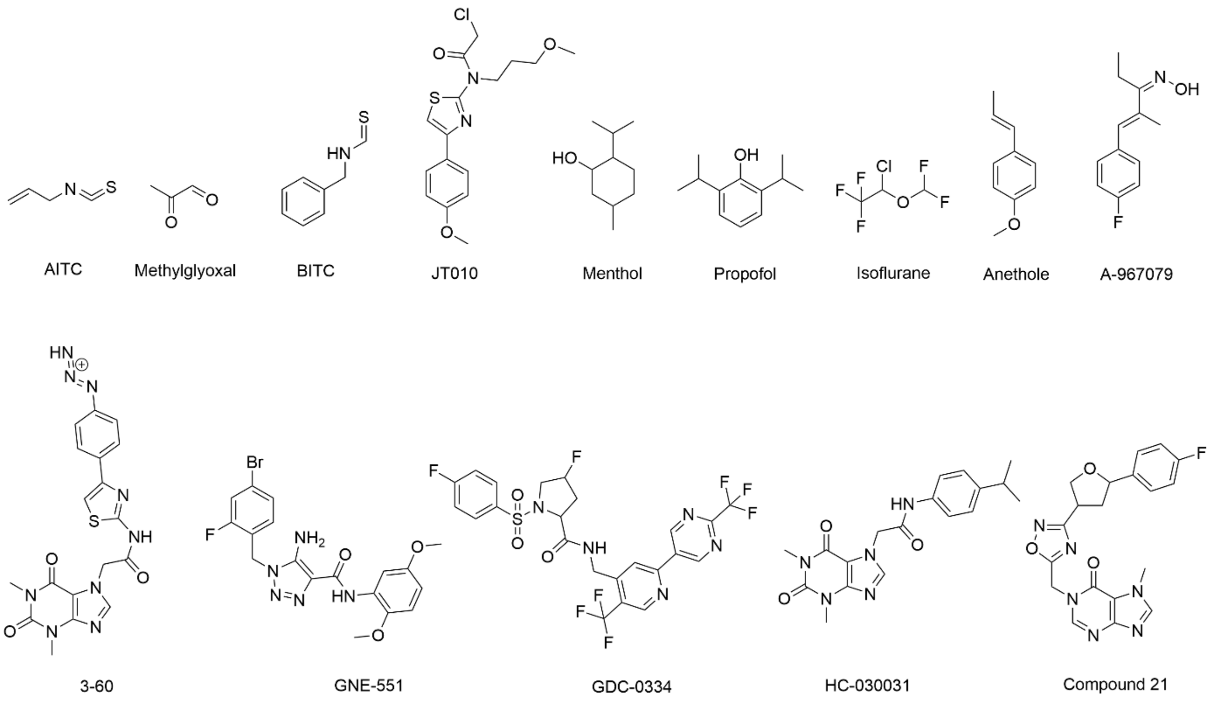

Figure S1. Structures of electrophilic and non-electrophilic TRPA1 modulators.

Table S1. Reported binding sites of electrophilic and non-electrophilic TRPA1 modulators

TRPA1 modulators	Mode of action	Binding site	Key residues for ligand activity/interaction	Reference
**AITC**	Electrophilic agonist (irreversible)	N-terminal cysteine residues	C621, C641, C665, K710[a]	(Bahia et al., 2016; Hinman et al., 2006), Reviewed in Meents et al. (2019)
**methylglyoxal**	Electrophilic agonist (reversible)	N-terminal cysteine residues	C621, C641, C665, K710[a]	(Eberhardt et al., 2012)
**BITC**	Electrophilic agonist (reversible)	N-terminal cysteine residues	C621[a,b], F612[a], Y680[a], I623[b]	(Suo et al., 2020)
**JT010**	Electrophilic agonist (irreversible)	N-terminal cysteine residues	C621[a,b], F612[a], Y680[a], T684[a,b], Y662[a], I623[a,b], C665[a], F669[a]	(Suo et al., 2020)
**menthol**	Non-electrophilic agonist	TM5	S873, T874, V875[a]	(Xiao et al., 2008)
**propofol**	Non-electrophilic agonist	TM5-PH1	S873, M912, M953[a]	(Ton et al., 2017)
**isoflurane**	Non-electrophilic agonist	TM5-PH1	S873, M912, M953[a]	(Ton et al., 2017)
**anethol**	Non-electrophilic agonist	TM5	S873, T874[a]	(Memon et al., 2019)
**A-967079**	Non-electrophilic antagonist	TM5-PH1	S873, T874, L881, F909, F944, V948, I950[a]	(Klement et al., 2013; Nakatsuka et al., 2013; Paulsen et al., 2015), Reviewed in Meents et al. (2019)
**GNE-551**	Non-electrophilic agonist	TM4-TM5-TM6	A836[b], Y840[a,b], F841[b], S887[b], Q940[a,b], S943[b], F947[b]	(Liu et al., 2021)
**GDC-0334**	Non-electrophilic antagonist Phase I clinical trial	TM5-PH1-TM6	-	(Balestrini et al., 2021)
**HC-030031**	Non-electrophilic antagonist	TM4-TM5 linker	N855[a]	(Gupta et al., 2016)
**Compound 21**	Non-electrophilic antagonist	pre-TM1, TRP-like domain and TM4-TM5 linker	H983, Q979, R852, Y711[b]	(Terrett et al., 2021)
**3-60**	Non-electrophilic antagonist	pre-TM1, TRP-like domain and TM4-TM5 linker	E854, N855, Y711 F853[b]	(Grieben et al., 2022)

[a] mutagenesis-based data; [b] cryo-EM data

Table S2. Summary of the effect of stilbenoids on TRPA1

Study	Method	Stilbenoids	Concentration	Observations
Yu 2013	patch clamp	-resveratrol<br>-PME[a]<br>-*trans*-stilbene	30 µm	-resveratrol inhibited AITC[b]/2APB[c]-induced mTRPA1 but not capsaicin[d]-induced rTRPV1<br>-PME inhibited capsaicin[d]-induced rTRPV1 but not AITC-induced mTRPA1<br>-*trans*-stilbene showed no effect on AITC[b]/2APB[c]-induced mTRPA1 or capsaicin-induced rTRPV1<br>-in DRG[e], pretreatment with resveratrol and PME (at 30 µM) suppressed AITC (at 300 µM) and capsaicin (30 nM) activities, respectively.<br>-resveratrol and PME alone at 30 µM did not induce any changes in membrane currents in HEK293 cells expressing mTRPA1 or rTRPV1, or in rat DRG neurons.<br>-pretreatment (topical) with resveratrol or PME (at 300 µM) was found to reduce paw flinches, but not paw licks, in rats induced by AITC or capsaicin, respectively.<br>-using resveratrol or PME alone did not elicit any inflammatory and painful reactions in rats.
Moilanen 2015	Fluo-3-AM<br>patch clamp	-resveratrol<br>-pinosylvin	-in Fluo-3-AM assay: 0.1, 1, 10, 30, 60 and 100 µm<br>-in patch clamp: 10, 30 and 100 µm	-pinosylvin in dose dependent manner (0.1-100 µm) inhibited AITC-induced Ca^{2+} influx (AITC, 50 µM) in HEK293 cells expressing hTRPA1 with IC_{50} 26.5 µm using a Fluo-3-AM assay.<br>-resveratrol and pinosylvin inhibited AITC[f]-induced hTRPA1 in dose dependent manner (3-100 µm) with IC_{50} 12.9 and 16.7 µm, respectively.<br>-resveratrol and pinosylvin activated hTRPA1 at 100 µm<br>-these compounds (10 mg/kg) eliminated AITC-induced edema using a mTRPA1-mediated acute inflammation model.
Nalli 2016	Fluo-4-AM	-resveratrol<br>-pinosylvin<br>-PME<br>-pterostilbene<br>-stilbenoid analogues	30 µm	-resveratrol and pinosylvin inhibited AITC[g]-induced rTRPA1 with IC_{50} 19.9 and 12.1 µm, respectively, but not capsaicin-induced hTRPV1<br>-resveratrol and pinosylvin did not activate rTRPA1 in transfected HEK293 cells<br>-PME inhibited capsaicin-induced hTRPV1 (IC_{50} 23.7 µM) and inhibited AITC[g]-induced rTRPA1 with IC_{50} 6.9 µm, also activated rTRPA1 with EC_{50} 3.5 µm<br>-pterostilbene activated rTRPA1 with EC_{50} 3.6 µm and inhibited AITC-induced rTRPA1 with IC_{50} 7.5 µm no effect on hTRPV1
Nakao 2017	Fluo-4-AM	-resveratrol<br>-pinosylvin<br>-PME<br>-stilbenoid analogues	30 µm	-pinosylvin evoked calcium influx in hTRPA1-expressing HEK293 cells<br>-resveratrol and PME activated hTRPA1 and inhibited AITC-induced hTRPA1

[a] pinosylvin monomethyl ether; [b] at concentration of 100 µM; [c] 2-aminoethoxy diphenyl borate at concentration of 400 µM; [d] at concentration of 20 nM; [e] dorsal root ganglion; [f] at concentration of 30 µM; [g] at concentration of 100 µM

Table S3. The cryo-EM structures of TRPA1 in the Protein Data Bank (PDB) (as of 30th June 2024)

Structure PDB ID	Year	Organism	Ligand	Pore state	Resolution	Reference
3J9P	2015	*Homo sapiens*	-	Partially closed	4.24 Å	(Paulsen et al., 2015)
6PQO	2020	*Homo sapiens*	JT010 covalent agonist	Partially closed	2.88 Å	(Suo et al., 2020)
6PQP	2020	*Homo sapiens*	BITC covalent agonist	Partially closed	3.06 Å	(Suo et al., 2020)
6PQQ	2020	*Homo sapiens* C621S mutant	-	Partially closed	2.81 Å	(Suo et al., 2020)
6V9V	2020	*Homo sapiens*	Iodoacetamide covalent agonist	Partially closed	2.6 Å	(Zhao et al., 2020)
6V9W	2020	*Homo sapiens*	-	Closed	3.1 Å	(Zhao et al., 2020)
6V9X	2020	*Homo sapiens*	-	Open	3.3 Å	(Zhao et al., 2020)
6V9Y	2020	*Homo sapiens*	-	Partially closed	3.6 Å	(Zhao et al., 2020)
6X2J	2020	*Homo sapiens*	GNE-551 agonist	Closed	3 Å	(Liu et al., 2021)
6WJ5	2021	*Homo sapiens*	GDC-0334 antagonist	Closed	3.6 Å	(Balestrini et al., 2021)
7JUP	2021	*Homo sapiens*	Compound 21 antagonist	Closed	3.05 Å	(Terrett et al., 2021)
7OR0/1	2022	*Homo sapiens*	3-60 antagonist	Partially closed	2.64 Å	(Grieben et al., 2022)
7YKR	2023	*Drosophila melanogaster*	-	State-1, closed	3.2 Å	(Wang et al., 2023)
7YKS	2023	*Drosophila melanogaster*	-	State-2, closed	3 Å	(Wang et al., 2023)

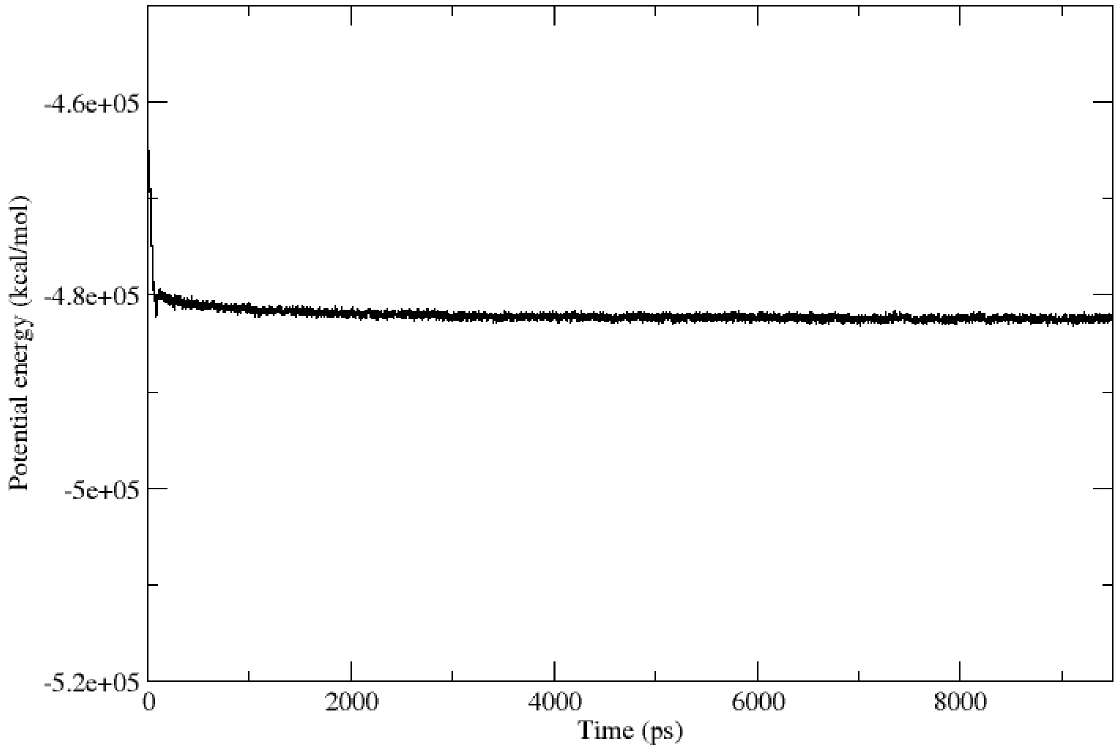

Figure S2. Potential energy of the molecular dynamics (MD) simulation system during the equilibration (500 ps) and the consequent 9-ns production simulation of human TRPA1 (PDB ID: 3J9P) using the Amber 16 MD simulation package

The stilbenoids in the HC-030031 binding pocket of hTRPA1

Since there is no experimental HC-030031–hTRPA1 complex available, we studied the stilbenoids binding to that antagonistic site based on the hTRPA1 cryo-EM structures with the analogous xanthine (cpd 3-60) and hypoxanthine (cpd 21) derivatives. The reference cpd 21 and the stilbenoids were docked to the cpd 21 site in the closed-state cryo-EM structure of hTRPA1 (PDB ID: 7JUP) and cpd 3-60 and the stilbenoids to the cpd 3-60 site in the intermediate-state hTRPA1 structure (PDB ID: 7OR0). Of note, the xanthine/hypoxanthine moiety of cpd 3-60 and cpd 21 share the same position, stacking strongly with Trp711, but then the rest of the compounds directs to different sides (subsites) of the pocket (Figure S3A, p. S9).

The predicted binding affinity of the stilbenoid compounds for the antagonist site (both subsites) was less strong (the more negative value means stronger interaction) than that for the A-967079 pocket when comparing their Prime/MM-GBSA ΔG-bind values (Table S4, p. S10). At the cpd 21 subsite, while the stilbenoids positioned similarly to cpd 21, they shared only one interaction with the reference (π-π stacking with Trp711). Although the stilbenoid glucosides formed more H-bond interactions with hTRPA1 than cpd 21, they did not demonstrate a comparable binding free energy to compound 21. At the cpd 3-60 subsite, all of the stilbenoids, except for isorhapontin, interacted with Trp711. However, only isorhapontin shared a similar position with cpd 3-60 and showed a better Prime/MM-GBSA ΔG-bind value compared to the other stilbenoids. None of the stilbenoids formed H-bond interactions with Asn855. It appears that the aglycone stilbenoids are notably small for this binding site, unable to fill either of the subsites. This is reflected in their less negative (weaker) binding free energy values at this site.

For comparison, we also docked HC-030031 into both subsites of this large antagonistic pocket. Even though HC-030031 aligned with cpd 21's position (Figure S3C) and mostly mimicked its interactions, it could only interact with Asn855 (a key amino acid for HC-030031 binding identified in mutational studies) in the cpd 3-60 subsite. The xanthine and acetamide moieties of HC-030031 and cpd 3-60 were well superimposed, forming interactions with Trp711 and Glu854 (bb) and Asn855, but the rest of molecules did not align (Figure S3B).

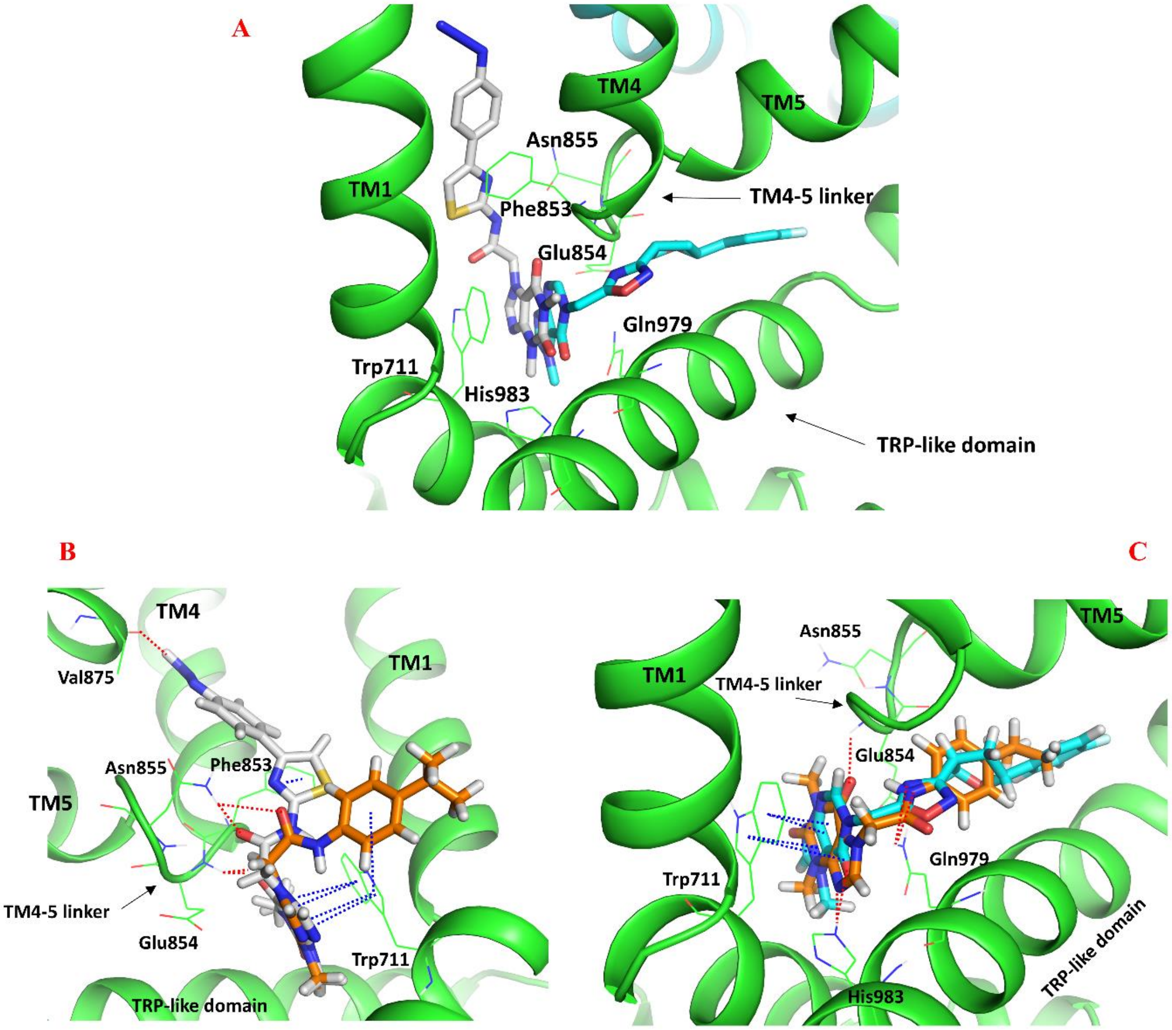

Figure S3. (A) Experimental binding poses of cpd 21 (cyan sticks) and cpd 3-60 (white sticks) in the HC-030031 pocket at their respective subsites of hTRPA1 (green and cyan cartoon; subunits with different colors) (PDB IDs: 7JUP and 7OR0 superimposed, the protein structure of 7JUP is hidden for clarity). (B) The docked pose of HC-030031 (orange sticks) compared to the experimental pose of cpd 3-60 (white sticks) (PDB ID: 7OR0). (C) The docked pose of HC-030031 compared to the experimental pose of cpd 21 (cyan sticks) (PDB ID: 7JUP). Atom color code: nitrogen – blue; oxygen – red; fluorine – light blue; sulphur – gold; hydrogen – white. The key residues (in lines) and the TM domains are labelled. Interaction color code (dashed lines): H-bond – red; π-π – blue.

Table S4. Docking results of the stilbenoids at the antagonistic HC-030031 binding site of hTRPA1 (PDB ID: 7JUP; 7OR0)

Compounds	Prime/MM-GBSA ΔG-bind (kcal/mol)[a]		Interactions H-bond[b]/ π–π interactions	
	cpd 21 subsite	cpd 3-60 subsite	cpd21 subsite	cpd 3-60 subsite
Resveratrol	-43.35	-44.87	Glu854, Arg852 (bb) /Trp711	Arg852 (bb), Lys704 (bb) /Trp711
Pinosylvin	-48.80	-44.61	Glu854 (bb), Ile858 (bb) /Trp711	Lys704 (bb) /Trp711
PME	-53.67	-44.60	Glu854 (bb), Ile858 (bb) /Trp711	Arg852 (bb) /Phe853, Trp711
Astringin	-59.04	-45.36	Glu854, Arg852 (bb), Arg975, Lys704 (bb) /Trp711	Glu854, Lys704 (bb) /Trp711
Isorhapontin	-67.25	-52.72	Glu854 (bb), Arg852 (bb), Met978 (bb), Gln979	Leu707 (bb), Gln979/Phe853
HC-030031	-77.09	-60.79	His983, Glu854(bb)/Trp711	Glu854 (bb), Asn855/Trp711
cpd 21	-83.18[c]	-	His983, Gln979/Trp711	-
cpd 3-60	-	-72.26[d]	-	Glu854 (bb), Asn855, Val875 (bb) /Phe853, Trp711

[a] To be able to compare the experimental complexes and the docking results at the cpd 21 and cpd 3-60 subsites, Prime/MM-GBSA binding free energies were calculated for all the docked complexes. [b] Hydrogen bond formed with the polar side chain atoms of the residue if not marked with bb (backbone atom). [c] Binding free energy of the experimental complex (PDB ID: 7JUP). [d] Binding free energy of the experimental complex (PDB ID: 7OR0).

The stilbenoids in GNE-551 binding pocket of hTRPA1

Since the in vitro study by Nakao et al. (Nakao et al., 2017) demonstrated that stilbenoids can indeed activate hTRPA1 channel and may subsequently inhibit the channel through a desensitization mechanism, we also explored the possibility that stilbenoids may interact with the other known agonistic site apart from the shared agonist/antagonist site of A-967079. Therefore, we docked the natural substances into the GNE-551 binding site using the same hTRPA1 structure as for the docking studies at the A-967079 site as there was no significant difference in docking scores/ interactions of GNE-551 in that structure compared with the original GNE-551–hTRPA1 complex structure (PDB ID: 6XJ2, see Table S5). Pinosylvin and PME exhibited similar binding poses, interacting with Gln940 and Phe841, whereas the pose of resveratrol was somewhat shifted to form an additional H-bond interaction with Ser943. Although astringin and isorhapontin were forming hydrogen bonds with many same residues (Table S5), their poses were noticeably distinct from each other. However, while all natural compounds were able to interact with at least some of the key residues important for agonist binding, astringin's orientation and position was closest to that of GNE-551, and its many hydrogen bonding interactions contribute especially to the Glide XP docking score (Figure 4E, F; Table S5). Further, the stilbenoids, specifically aglycons, are not sufficiently bulky to make use of all the possible interactions in the GNE-551 pocket, which is also reflected in the free energy of binding values compared to those in the A-967079 binding pocket. Based on the interactions, docking scores, and the binding free energy values, it appears that the stilbenoids show a higher affinity to agonistic/antagonistic pocket (TM5-PH1-TM6) than the agonistic pocket (GNE-551 binding site).

Table S5. Docking results of the stilbenoids at the agonistic GNE-551 binding site of the intermediate-state hTRPA1 (PDB ID: 3J9P, MD frame saved at 7 ns)

Compounds	XP GScore (kcal/mol)	Prime/MM-GBSA ΔG-bind (kcal/mol)	H-bond	π-π interaction
Resveratrol	-5.73	-53.00	Gln940, Ser943	Phe841
Pinosylvin	-4.23	-44.90	Gln940	Phe841
PME	-4.34	-44.10	Gln940	Phe841
Astringin	-7.81	-52.71	Gln940, Ser943, Asn798, Tyr799 (bb), Met801 (bb)	-
Isorhapontin	-4.04	-43.05	Tyr840, Ser943, Asn798, Tyr799 (bb)	-
GNE-551	-5.25/-5.63[a]	-66.83/-56.9[a]/ -68.55[b]	Tyr840[c]	Tyr840, Phe841[c]

[a] The corresponding values of the redocked GNE-551 in the original GNE-551–hTRPA1 cryo-EM complex (PDB ID: 6X2J). [b] Binding free energy value for the original GNE-551–hTRPA1 cryo-EM complex. [c] Same interactions when redocked to PDB ID: 6X2J; see Table S1 for the interactions of the experimental complex).

Table S6. Docking results of resveratrol at the A-967079 (antagonist) and GNE-551 (agonist) binding sites of intermediate-state hTRPA1 (PDB ID: 3J9P, MD frame saved at 7 ns) and the rTRPA1 model

**Compounds**	**hTRPA1 binding site**		**rTRPA1 binding site**	
	**A-967079**	**GNE-551**	**A-967079**	**GNE-551**
	**Prime/MM-GBSA ΔG-bind (kcal/mol)**			
**Resveratrol**	-54.97	-53.00	-56.78	-44.85
**A-967079**	-44.22	-	-55.59	-
**GNE-551**	-	-66.83	-	-55.68

Table S7. Binding free energy of resveratrol at the A-967079 (antagonist) and GNE-551 (agonist) binding sites of intermediate-state hTRPA1 (PDB ID: 3J9P, MD frame saved at 7 ns) and the rTRPA1 model at the end of 400-ns parallel MD simulations.

Compounds	GNE-551 binding site		A-967079 binding site	
	hTRPA1	rTRPA1	hTRPA1	rTRPA1
	**Prime/MM-GBSA ΔG-bind (kcal/mol)** after MD simulation			
**Resveratrol**	-53.00 (initial)[a]	-44.85 (initial)[a]	-54.97 (initial)[a]	-56.78 (initial)[a]
	-52.26	-43.13	-61.21	-54.8
	-51.88	-49.86	-53.05	-55.53
	-58.3	-29.07	-57.42	-51.61
		-39.34		
		-50.34		
**GNE-551**	-68.55 (initial)[a,b]	-55.68 (initial)[a]	-	-
	-67.14 [b]	-58.41		
	-64.19 [b]	-66.00		
	-63.67 [b]	-38.99		
		-41.08		
		-49.32		

[a] Binding free energy value of the compounds before 400-ns parallel MD simulations. [b] Binding free energy value of the original GNE-551–hTRPA1 cryo-EM complex (PDB ID: 6X2J).

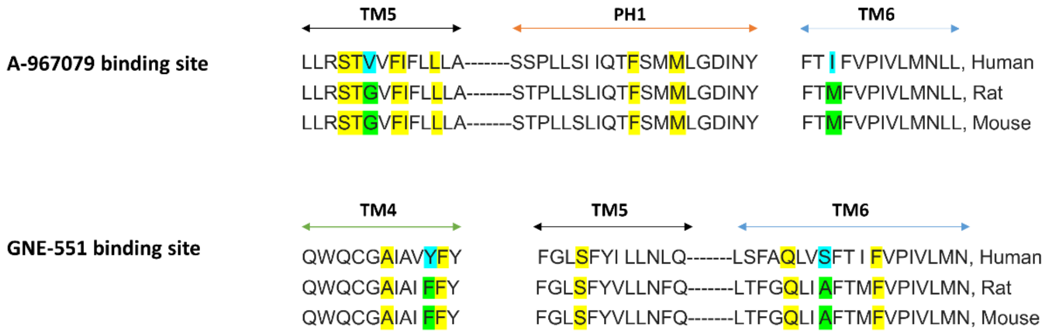

Figure S4. Sequence alignment of human, rat and mouse TRPA1 for A-967079 and GNE-551 binding sites.

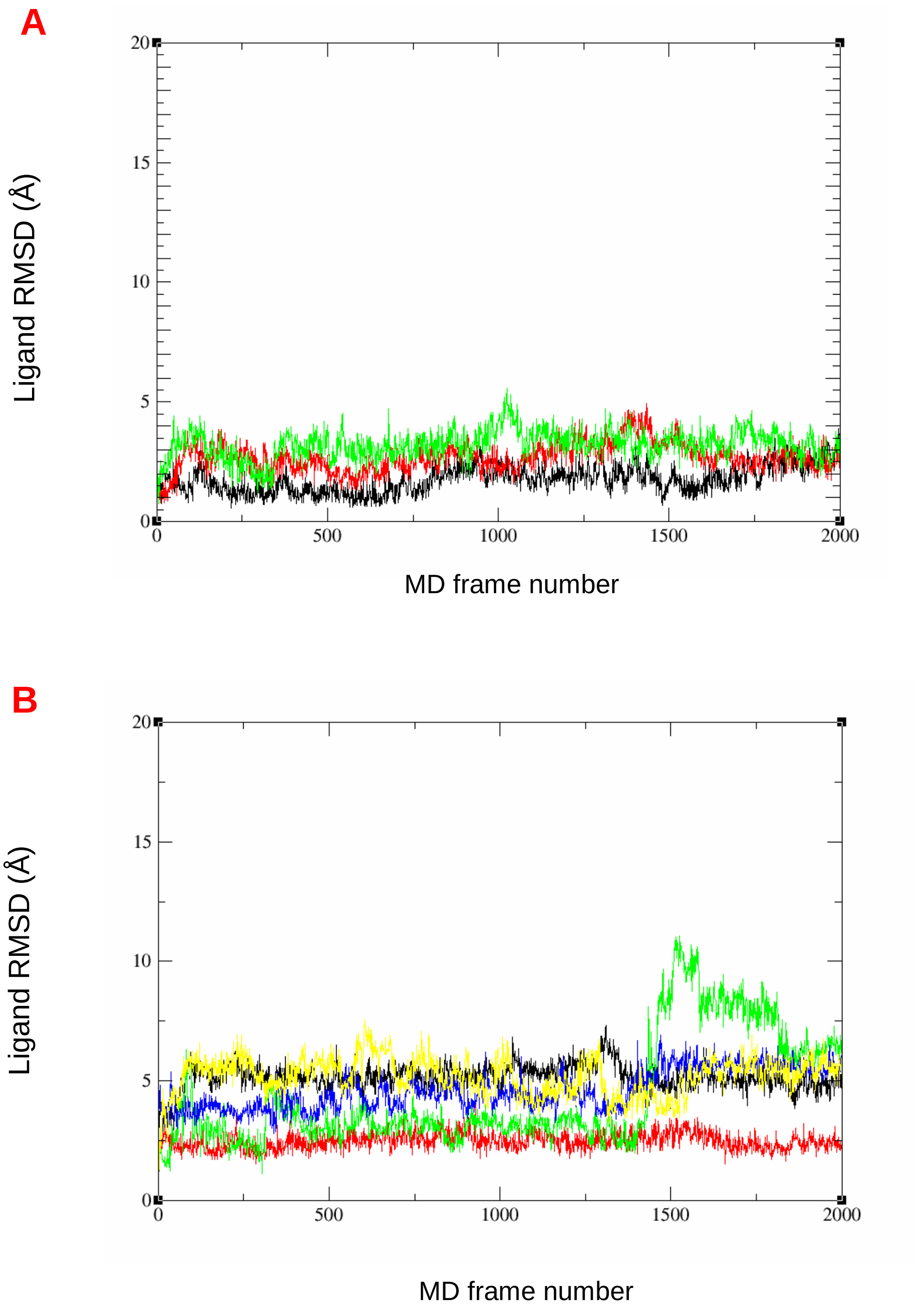

Figure S5. Ligand RMSD plots of GNE-551 in its binding site at (A) hTRPA1 (PDB ID: 6X2J) and (B) the rTRPA1 model during repeated (parallel) 400-ns molecular dynamics (MD) simulations. Every color represents the ligand RMSD during an individual simulation. Snapshots of the MD trajectory (frames) were saved at every 200 ps.

A

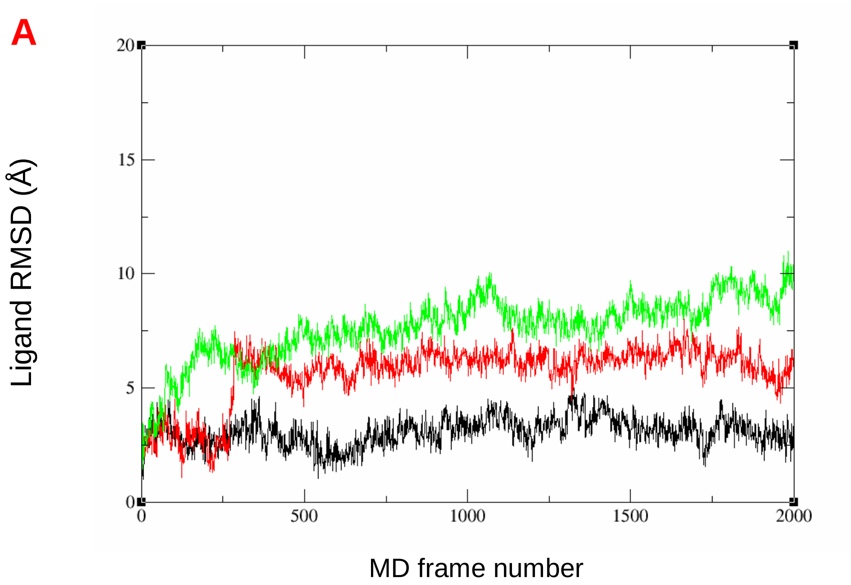

B

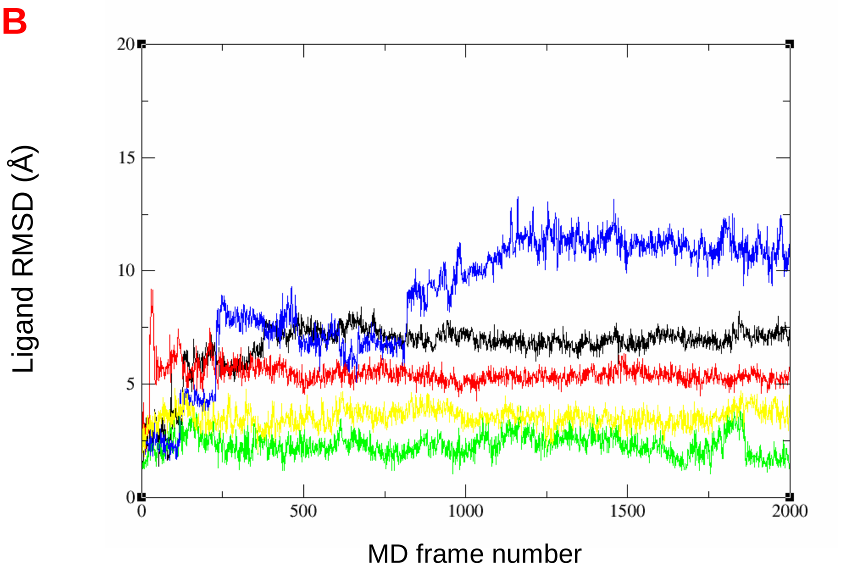

Figure S6. Ligand RMSD plots of resveratrol in the GNE-551 pocket at (A) hTRPA1 (refined intermediate state; PDB ID: 3J9P) and (B) the rTRPA1 model during repeated (parallel) 400-ns molecular dynamics (MD) simulations. Every color represents the ligand RMSD during an individual simulation. Snapshots of the MD trajectory (frames) were saved at every 200 ps.

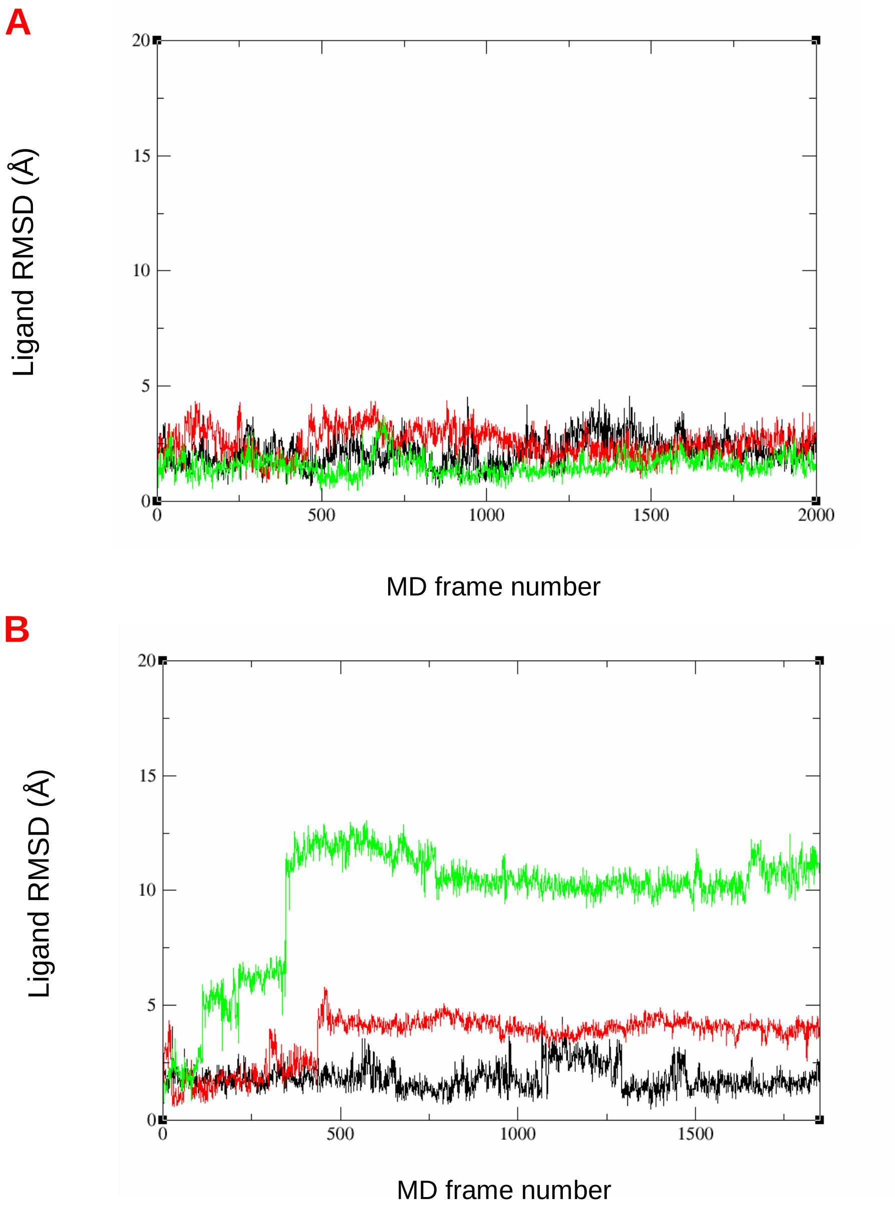

Figure S7. Ligand RMSD plots of resveratrol in the A-967079 pocket at (A) hTRPA1 (refined intermediate state; PDB ID: 3J9P) and (B) the rTRPA1 model during repeated (parallel) 400-ns molecular dynamics (MD) simulations. Every color represents the ligand RMSD during an individual simulation. Snapshots of the MD trajectory (frames) were saved at every 200 ps.

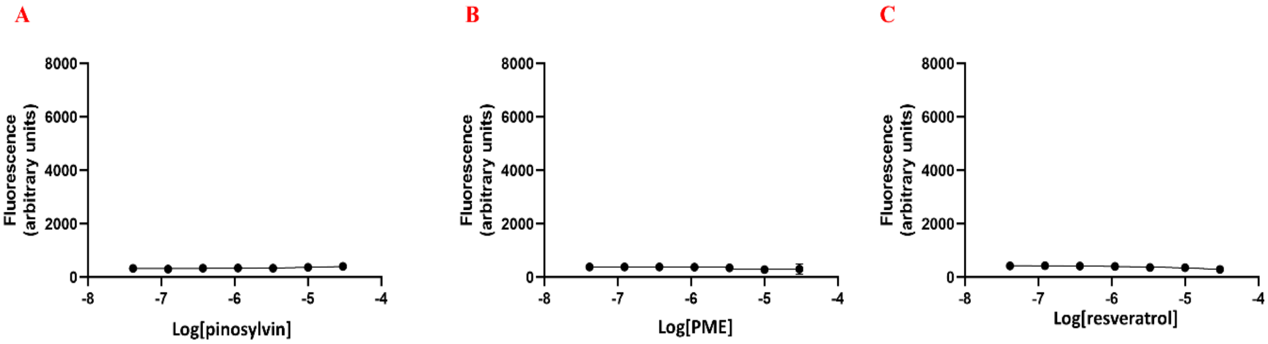

Figure S8. The FLIPR™ assay shows (in)activity of pinosylvin (A), PME (B) and resveratrol (C) in non-transfected HEK293 cells.
